# Low-level Blood Multiple Metals Exposure from Informal Jewelry Welding Alters Metabolomic and Lipidomic Profiles in Brazilian Women

**DOI:** 10.1007/s12011-026-05096-4

**Published:** 2026-04-16

**Authors:** Alda Neis Miranda de Araújo, Vinicius Guimarães Ferreira, Isabelle Nogueira Leroux, Danilo Cardoso de Oliveira, Andreia M. Porcari, Kelly Polido Kaneshiro Olympio, Nilson Antônio de Assunção

**Affiliations:** 1 Graduate Program in Translational Medicine, Paulista School of Medicine, Department of Medicine, Federal University of São Paulo, São Paulo, Brazil; 2 Departament of Environmental Health, School of Public Health, University of São Paulo, São Paulo, Brazil; 3 The Human Exposome Research Group/Expossoma e Saúde do Trabalhador – eXsat, School of Public Health, University of Sao Paulo, São Paulo, Brazil; 4 MS4Life Laboratory of Mass Spectrometry, Health Sciences Graduate Program, São Francisco University, Bragança Paulista, Brazil; 5 Department of Chemistry, Institute of Environmental, Chemical and Pharmaceutical Sciences Federal University of São Paulo, Diadema, São Paulo, Brazil

**Keywords:** Exposure assessment, Heavy metals, Metabolomics, Lipidomics, Occupational exposure

## Abstract

Occupational exposure to toxic and potentially toxic elements (PTEs) remains a significant public health concern due to its potential to disrupt human metabolic processes. This study investigated metabolic and lipidomic alterations associated with blood PTE levels in Brazilian women engaged in informal, home-based jewelry welding. A total of 64 participants were included: a welder group (*n* = 36) and a control group (*n* = 28). Blood PTE concentrations were quantified using inductively coupled plasma mass spectrometry (ICP-MS), and plasma metabolites were profiled using untargeted MS-based metabolomics. Significant between-group differences were observed for arsenic (As; *p* = 0.01), cadmium (Cd; *p* = 0.01), and lead (Pb; *p* < 0.001). Overall, 190 metabolites and 35 lipids were identified at MS¹ and MS² levels. Of these, 53 metabolites and 11 lipids exhibited significant correlations with PTE levels, including phenylalanine (Pb, Cd), acetylcarnitine (As), Sudan I (Cd), hexyl glucoside (Cd, Pb), and MG 22:5 (As, Pb). Enrichment analysis revealed alterations in purine and porphyrin metabolism in the metabolomics dataset, whereas lipidomics indicated disruptions in glycerolipid metabolism, dietary lipid digestion, and ABC transporter-related pathways. Notably, these associations were observed even at low blood PTE levels, indicating biologically relevant molecular alterations and underscoring the need for improved occupational protections in informal working environments.

**Supplementary Information:**

The online version contains supplementary material available at 10.1007/s12011-026-05096-4.

## Introduction

Chemical exposures, regardless of origin, have become an unavoidable aspect of modern life. Food, water, and air are increasingly recognized as sources of contamination [1–3]. The scientific community has actively emphasized the associated risks and evaluated contaminant levels across various sources. However, one important source of exposure is frequently overlooked: the work environment. Emerging evidence indicates growing risks to workers from occupational exposure to toxic and potentially toxic elements (PTEs) [4, 5]. Assessing these exposures is essential for understanding occupational disease causation, prevention, risk perception, working conditions, and environmental monitoring [4, 5].

As contaminant profiles vary considerably according to environmental conditions and factors specific to each work activity, high-risk occupational environments should be studied individually [6, 7]. This approach enables the identification of major contaminants, assessment of exposure levels, and evaluation of potential adverse health effects [8, 9].

Among such environments, home-based and informal work, such as jewelry welding in Brazil, demand particular attention due to the frequent lack of protective measures, regulatory oversight, and safe handling guidelines, as well as the uncertain origin of raw materials [10, 11]. Exposure to welding fumes represents a major concern, as approximately 3% of the economically active population is exposed to this contaminant [12, 13]. These fumes contain multiple potentially toxic elements (PTEs), including nickel (Ni), lead (Pb), tin (Sn), copper (Cu), arsenic (As), mercury (Hg), chromium (Cr), manganese (Mn), antimony (Sb), and cadmium (Cd) [12, 14–16].

Previous studies from our group have characterized PTE concentrations in this occupational environment. In study [17], elevated concentrations of these elements were measured in the breathing zones of welders, exceeding environmental exposure limits established by the Agency for Toxic Substances and Disease Registry (ATSDR) for Mn, Ni, Zn, Cd, and Pb, as well as Occupational Safety and Health Administration (OSHA) limits for Cu, Zn, Cd, and Pb. In the same study, concentrations of Mn, Ni, and Cd also exceeded the minimum risk level (MRL) for chronic inhalation [17]. Another study [18] reported elevated urinary Cd concentrations in female welders. Furthermore, study [11] observed correlations between the concentrations of Cr, Pb, and As in household dust where welding activities occur and in the blood of children living in these environments. In addition to the PTEs found in blood, urine, and air, investigations using a portable X-ray analyzer detected lead in welding wires and cadmium in welding powder [11]. Moreover, study [18] identified Cd and Pb in jewelry pieces produced within this productive arrangement. Exposure to these substances can result in acute effects (e.g., skin and respiratory irritation) and chronic outcomes across multiple organ systems, including lung cancer and respiratory, hepatic, renal, neurological, and cardiovascular disorders [6, 19–25].

The exposome encompasses the totality of exposures encountered throughout life, including external environments, occupational environments, behaviors, diet, and endogenous biological processes [4, 26, 27]. Exposomics employs multi-omics technologies to characterize internal biochemical responses to complex exposure histories. It captures molecular signatures across biological layers (e.g., DNA/RNA, proteins/enzymes, and metabolites), thereby elucidating the biological effects of environmental and occupational exposures [26, 27]- [28].

Unlike other omics disciplines, metabolomics provides an accurate reflection of both endogenous and exogenous biochemical disturbances, making it a powerful tool for understanding how environmental interactions influence metabolic pathways [29, 30]. Plasma metabolomic and lipidomic profiles represent the current biochemical state of cells, making them particularly suitable for detecting early biological effects of environmental and occupational exposures [31, 32].

This study aims to characterize metabolomic and lipidomic alterations associated with occupational exposure to low levels of PTEs in women jewelry welders. By combining these omics approaches within a single biological matrix, we seek to elucidate the molecular mechanisms underlying exposure-related health risks in an informal occupational environment.

## Methods

### Study Population

This study was approved by the Research Ethics Committee (Comitê de Ética em Pesquisa – CEP, in Portuguese) of the School of Public Health, University of São Paulo (FSP/USP) (CAAE No. 41965115.0.0000.5421) and by the CEP of the Federal University of São Paulo (UNIFESP) (CAAE No. 04354818.0.1001.5505). All individuals voluntarily agreed to participate in the study and signed a standardized informed consent form.

Study participants were selected through non-probability sampling, with convenience sampling applied to exposure groups. Recruitment was conducted with support from the Municipal Health Department of Limeira and Primary Health Care Units (UBS) of the Brazilian Unified Health System (SUS, Sistema Único de Saúde, in Portuguese). Contact with informal workers was facilitated by Community Health Agents (ACS, Agentes Comunitários de Saúde, in Portuguese) from each neighborhood, affiliated with the Family Health Strategy (ESF, estratégia de saúde da família, in Portuguese). All households visited by ACS and identified as engaging in home-based jewelry production were invited to participate in the study.

As jewelry welding in this context is predominantly performed by women, only women aged 18 years or older were included. Participants were divided into two groups based on occupational exposure: the exposed group comprised women performing jewelry welding at home, while the control group consisted of women with no jewelry production activities or history of occupational chemical exposure. A total of 64 women participated in the study (36 exposed, 28 controls), with mean ages of 36.2 and 36.1 years, respectively.

Control households were identified from exposed workers’ residences: researchers walked clockwise along the street, facing the roadway, and selected the fourth house [17]. Eligible households were those where no resident reported engagement in jewelry or fashion jewelry production, either at home or in private companies. In cases of refusal or ineligibility, researchers proceeded to the next household following the same procedure. All inclusion criteria were verified by the research team prior to participant enrollment. Baseline demographic and clinical characteristics of the exposed and control groups (including BMI, education/socioeconomic status, smoking, alcohol consumption, and metabolic profile) were described previously [33].

#### Sample Collection

Blood collection followed standardized pre-analytical protocols. Participants were instructed to fast for 8 h, abstain from physical exercise for 48 h prior to collection, and avoid alcohol consumption during the preceding week. Venous blood samples were collected by trained phlebotomists between October and November 2019.

Plasma for metabolomics and lipidomics analyses was collected in 6 mL Vacutainer^®^ tubes containing lithium heparin. Immediately after collection, tubes were transported on ice from Limeira, São Paulo, to the Laboratory for Human Exposure to Environmental Contaminants (LEHCA). Samples were centrifuged at 2,000 × g for 10 min at 4 °C to obtain plasma, which was aliquoted into Eppendorf tubes and stored at − 80 °C until analysis.

For determination of toxic and potentially toxic elements (PTEs: As, Cd, Cr, Cu, Hg, Mn, Ni, Pb, Sb, Sn, and Zn), 6 mL of whole blood was collected in trace element‑free Vacutainer^®^ tubes containing heparin. Samples were transported on ice in insulated boxes to the laboratory and stored in an ultrafreezer at − 80 °C until analysis.

### Toxic and Potentially Toxic Elements determination

Blood concentrations of toxic and potentially toxic elements (PTEs: As, Cd, Cr, Cu, Hg, Mn, Ni, Pb, Sb, Sn, and Zn) were determined using a triple quadrupole inductively coupled plasma mass spectrometer (TQ-ICP-MS; Thermo Fisher Scientific, Bremen, Germany). A 200 µL aliquot of each blood sample was diluted with a solution containing Triton X-100 and nitric acid. An internal standard solution (1 mL) containing yttrium (Y), gallium (Ga), iridium (Ir), and terbium (Tb) was added to each sample. All samples were prepared using high‑purity deionized water (resistivity = 18.2 MΩ cm at 25 °C). Analyses were performed in triplicate, including analytical blanks.

Quality control was ensured through calibration curves (R² > 0.999) and analysis of the certified reference material (Seronorm^®^ TE Whole Blood Level II) after every 15 samples. Method performance showed 95–105% recovery, limits of detection (LODs) of 0.01–0.5 µg/L, limits of quantification (LOQs) of 0.03–1.5 µg/L, and coefficient of variation (CV) < 5%. Elemental determinations were performed using TQ-ICP-MS operated with argon, helium, and oxygen in the collision/reaction cell under optimized parameters. Full analytical details are described in our previous publication [33, 34].

### Plasma Metabolite and Lipid Extraction

Metabolites and lipids were extracted from plasma using the Folch method [35]. Plasma samples were thawed on dry ice, homogenized, and 30 µL aliquoted into Eppendorf tube. Methanol (MeOH, 225 µL), chloroform (450 µL), and ultrapure water (187.5 µL) were added sequentially with vortexing after each addition. Samples were incubated on a shaker for 1 h, centrifuged at 10,000 rpm for 5 min, and left at room temperature for 15 min. The aqueous phase (metabolites) and organic phase (lipids) were then collected into new tubes.

Following phase separation, Quality Control (QC) aliquots were prepared from 15 µL pooled from each individual sample, homogenized by vortexing for 15 s in a 5 mL centrifuge tube, and analyzed throughout batches to verify instrumental repeatability. Blank samples (100 µL water replacing plasma) underwent identical extraction for both metabolomic and lipidomic analyses. Extracts were dried at room temperature using a nitrogen evaporator and stored at − 80 °C until analysis.

### Mass Spectrometry (LC-MS/MS)

The extracts were resuspended in 200 µL of 50% methanol/acetonitrile (3:1, v/v) and filtered through a 0.22 μm membrane filter. Analyses were performed using a liquid chromatography system (1290 Infinity II UHPLC, Agilent Technologies) coupled to a QqTOF high-resolution mass spectrometer (Impact HD, BrukerDaltonics GmbH, Germany).

For metabolomic analyses, separation was performed on a Zorbax Eclipse XDB C18 column (100 × 3.0 mm i.d., 3.5 μm; Agilent Technologies). The LC gradient employed 0.1% formic acid in water (solvent A) and 0.1% formic acid in acetonitrile (solvent B), as detailed in Supplementary Table 1 (Online Resource 1).

For lipidomic analyses, separation was performed on a reversed-phase Eclipse SDB-C18 column (100 × 3.0 mm, 3.5 μm). The mobile phase consisted of 5 mM ammonium formate in water (solvent A) and a methanol/isopropanol mixture (MeOH: IPA, 85:15, v/v) containing 5 mM ammonium formate (solvent B). The elution gradient is described in Supplementary Table 2 (Online Resource 1).

For both methods, the column temperature was maintained at 40 °C, flow rate at 0.4 mL/min, and the injection volume was 2 µL. Total run times were 30 min for metabolomics and 28 min for lipidomics.

Main MS parameters included nebulizer gas pressure of 4.0 bar, drying gas flow of 8 L/min, and source temperature of 180 °C. Collision-induced dissociation (CID) used 5 eV collision cell energy, with full-scan mass range of 50–1300 m/z. Samples were analyzed in positive (4500 V) and negative (3000 V) electrospray ionization modes, with MS/MS data acquired via data-dependent acquisition (DDA); collision RF ranged from 200.0 to 550.0 Vpp, ion transfer time 50.0–90.0 µs (50.0% each), funnel RF 1 and RF 2 at 250.0 and 150.0 Vpp, hexapole RF at 50.0 Vpp, and quadrupole ion energy at 5.0 eV with 6.0 µs pre-pulse storage.

### Statistical Analysis

#### Toxic and Potentially Toxic Elements Data Analysis

To assess differences in blood concentrations of the 11 PTEs Student’s t-test or Mann–Whitney U test was applied, based on each variable’s distribution. Missing values were imputed using feature-wise K-nearest neighbors (KNN) algorithm. Descriptive statistics included geometric mean, standard deviation, 95% confidence interval, and 95th percentile for both groups.

For correlation analyses, PTE data underwent Pareto scaling to address heteroscedasticity and prevent dominance by high-variance/abundance metals. Spearman’s correlation coefficients were calculated between PTE concentrations and metabolite levels, considering |ρ| > 0.30 and *p* < 0.05 as relevant.

All data processing and statistical analyses were conducted in Python using Pandas, SciPy, and Scikit-Learn libraries. Data visualization, including boxplots, heatmaps, and chord diagrams, was carried out with Plotly and Matplotlib.

#### Omics Data Analysis

**Fig. 1:**
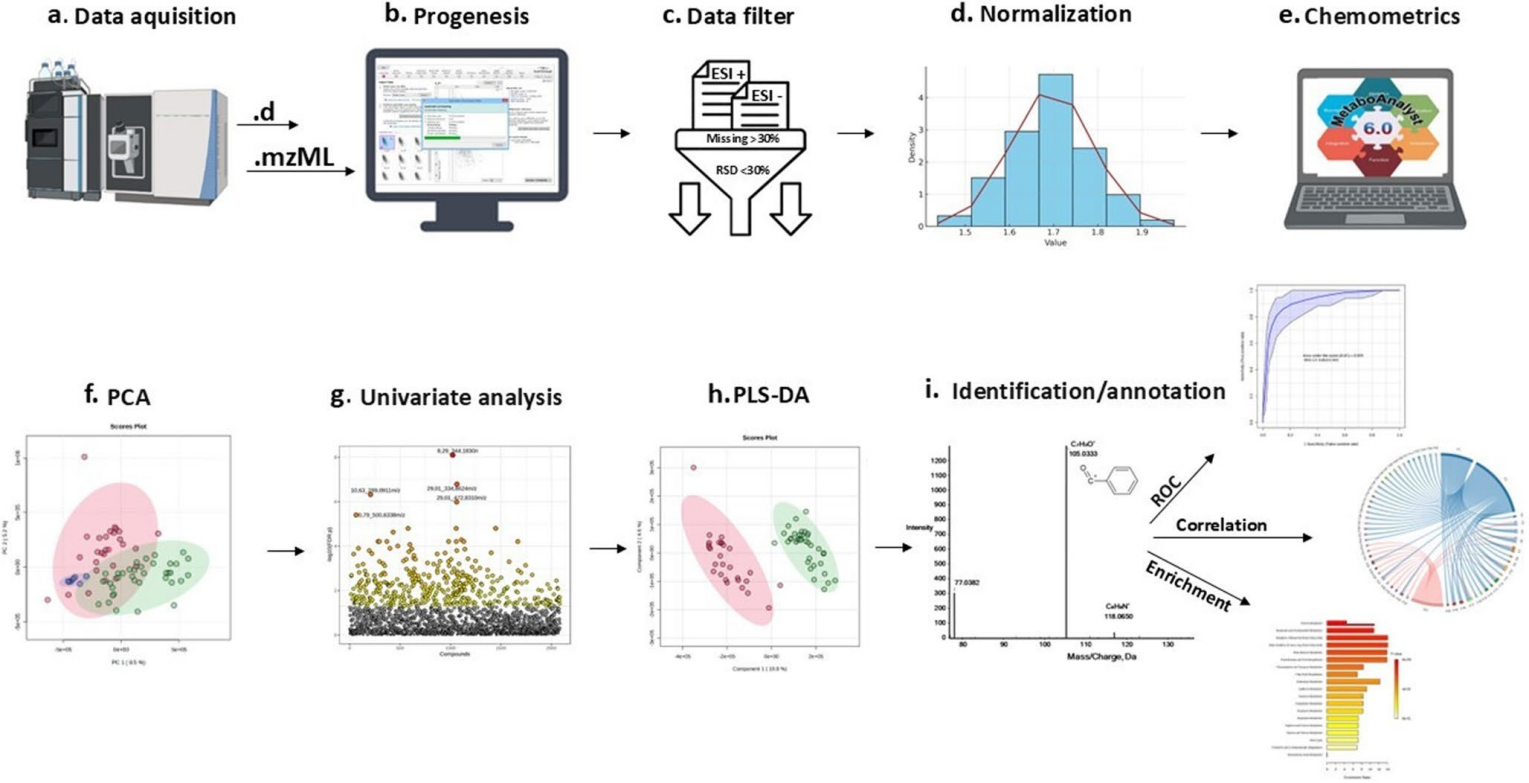
Schematic workflow of data processing, statistical analysis, and metabolite/lipid identification, enrichment analysis

Data processing was performed as illustrated in Fig. 1. For statistical analysis, data from both positive and negative ionization modes were integrated. Features with > 30% missing values were excluded, and remaining features with relative standard deviation (RSD) calculated in quality control (QC) samples [36] were retained to minimize analytical instability.

For metabolomics data, locally weighted scatterplot smoothing (LOWESS) normalization [37] corrected signal drift across the sequence based on QC samples. In contrast, lipidomics data underwent sum normalization in MetaboAnalyst, as stable QC metrics (low RSD) indicated no run-order drift; global sum normalization addressed between-sample signal differences. Post-normalization, only features with RSD < 25% in QC samples were retained for chemometric analyses.

Statistical analyses were performed using the MetaboAnalyst 6.0 platform [38]. Missing values were imputed using KNN algorithm [39, 40], followed by Pareto scaling [41]. An unsupervised principal component analysis (PCA) was conducted to assess samples clustering within each study group and to verify QC clustering.

Univariate analyses were performed using Student’s t-test (*p* < 0.05), with false discovery rate (FDR ≤ 0.05) adjustment for multiple testing, to select features showing greatest group differences. These features were used as input for multivariate modeling via partial least squares-discriminant analysis (PLS-DA) to assess group separation [25].

Finally, features were identified at both MS^1^ and MS^2^ confidence levels [42]. Receiver Operating Characteristic (ROC) curve analyses were then performed for the PLS-DA model using only identified features.

#### Features Identification

Spectra were processed separately in positive and negative ionization modes according to previously established conditions. For feature identification, positive mode adducts considered were [M + H]⁺, [M + NH₄]⁺, [M + Na]⁺, [M + H-H₂O]⁺, [M + ACN+H]⁺, and [M + ACN+Na]⁺. In negative mode adducts evaluated were [M-H]⁻, [M+HCOO]⁻, [M + Cl]⁻, and [M-H-H₂O]⁻.

Identification of statistically significant molecular features was conducted using the Progenesis™ QI v2.4 software (Waters Corporation™ © Nonlinear Dynamics, Newcastle, United Kingdom). Acquired spectra were compared with external libraries in.sdf format, including the Human Metabolome Database (http://www.hmdb.ca/metabolites), the MassBank of North America (MoNA, https://mona.fiehnlab.ucdavis.edu/), and LIPIDMAPS (http://www.lipidmaps.org/). Identification criteria included exact mass (mass error < 5 ppm), fragmentation pattern (mass error < 10 ppm), isotopic similarity, and biological plausibility of proposed metabolites.

Following feture identification, metabolite and lipid enrichment analyses were carried out in MetaboAnalyst 6.0 using the list of identified compounds. The *Homo sapiens* library from the Small Molecule Pathway Database (SMPDB) was used as the reference background.

## Results

### Toxic and Potentially Toxic Elements

Concentrations of PTEs determined in blood samples are presented in Table 1. Statistically significant differences between exposed and control groups were observed for As (*p* = 0.01), Cd (*p* = 0.01), and Pb (*p* < 0.001). Blood metal concentrations in welders were previously compared with national (Brazilian biomonitoring studies) and international reference distributions in a previous publication by our group [33].

**Table 1:** Geometric Mean (GM) and 95% confidence interval (CI) of toxic and potentially toxic elements (PTEs) measured in blood samples from exposed and control groups in Limeira, São Paulo, Brazil, 2019

Exposed group			Control group			
PTE µg/L	GM (CI 95%)	95th percentile	GM (CI 95%)	95th percentile	*p*-value	International benchmark
As	0.45 (0.38–0.53)	1.06	0.38 (0.25–0.56)	5.36	0.01*	NA
Cd	0.5 (0.33–0.74)	3.32	0.18 (0.10–0.30)	1.76	0.01*	A
Cr	1.07 (0.85–1.34)	4.21	1.10 (0.86–1.40)	4.99	0.69	NA
Cu (µg/L × 10³)	1.10 (1.03–1.17)	1.43	1.16 (1.08–1.25)	1.66	0.39	NA
Hg	1.11 (0.96–1.27)	2.17	1.07 (0.84–1.35)	4.18	0.32	NA
Mn	8.12 (7.09–9.28)	13.73	7.39 (6.47–8.43)	11.83	0.28	NA
Ni	1.09 (0.51–2.28)	5.83	1.55 (0.76–3.16)	5.27	0.96	NA
Pb (µg/dL) ^#^	1.74 (1.34–2.24)	4.65	0.94 (0.76–1.14)	1.90	< 0.001*	B
Sb	2.13 (2.00–2.25)	2.81	2.16 (2.01–2.32)	2.85	0.76	NA
Sn	0.83 (0.72–0.94)	1.31	0.86 (0.72–1.02)	1.37	0.46	NA
Zn (µg/L × 10³)	4.33 (3.98–4.72)	6.14	4.36 (3.94–4.83)	5.99	0.81	NA

* Statistically significant differences between groups (*p* < 0.05)

^#^ Pb values are reported in µg/dL as originally measured

**NA**: No internationally harmonized World Health Organization (WHO) and OSHA blood benchmark for direct comparison

**A**: For Cadmium (Cd): Occupational action framework: Under OSHA’s cadmium standard, medical surveillance actions are tied to target values for cadmium in blood (CdB: 5, 10, 15 µg/L) and to urinary Cd and β2-microglobulin criteria [43]

**B**: For lead (Pb): Public-health guidance: WHO notes that no safe blood lead concentration has been identified [44, 45]. Occupational/surveillance references: Brazil’s NR-7 lists Pb-S = 60 µg/100 mL as an occupational biological indicator (IBE/SC) [46], and the National Institute for Occupational Safety and Health (NIOSH) Adult Blood Lead Epidemiology and Surveillance (ABLES) program uses BLL ≥ 5 µg/dL as the reference value for an elevated adult blood lead level for surveillance [47, 48]. *These are action/surveillance references and do not represent universal “safe” thresholds*

### Omics Results

As previously described for this cohort [33], no significant differences were found between exposed and control groups regarding education, alcohol consumption, smoking, BMI categories, or baseline metabolic profile (insulin, glucose, total and HDL/LDL cholesterol, triglycerides, HOMA-IR; all *p* > 0.05).

#### Metabolomics

Data processing yielded 4,275 and 4,272 features in the positive and negative ionization modes, respectively. After normalization, merging, and filtering, 3,475 features were retained for chemometric analysis. PCA showed tight QC clustering and separation trends between exposed and control groups (Supplementary Fig. 1a, Online Resource 2), supporting analytical reproducibility and suggesting exposure-related metabolomic differences. PCA was performed on the combined dataset including control, exposed, and QC samples.

**Fig. 2:**
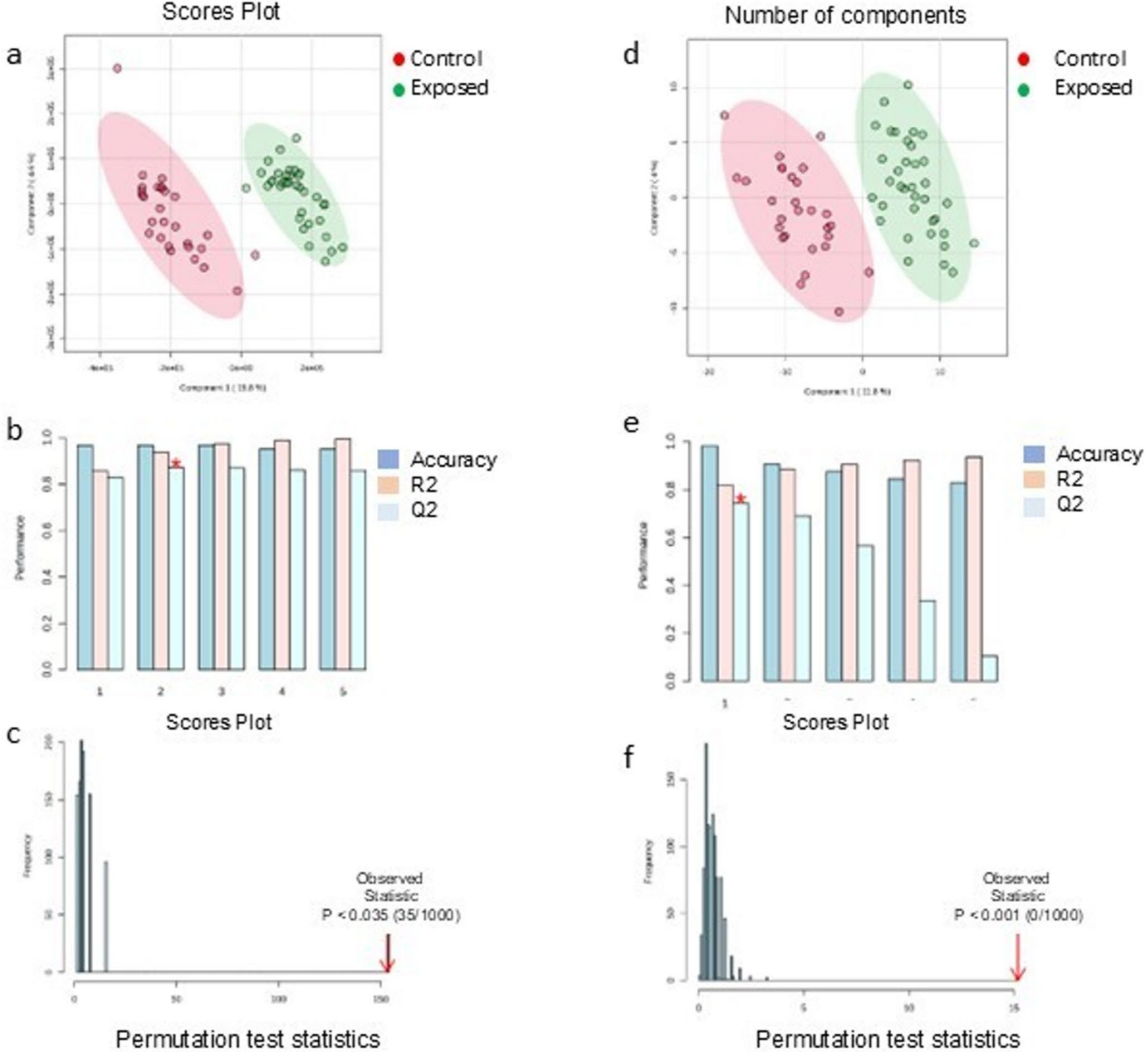
Scores plot from PLS-DA analysis illustrating separation between control (red) and exposed (green) groups. Left panel (**A**–**C**): metabolomics results for statistically significant features. Right panel (**D**–**F**): lipidomics results for statistically significant features. (A, D) Score plots showing distinct group separation based on PLS-DA components. (B, E) Cross-validation of PLS-DA models: metabolomics (B) shows R² = 0.90 and Q² = 0.90; lipidomics (E) shows R² = 0.80 and Q² = 0.70. (C, F) Permutation tests for model validation (*p* < 0.05)

Univariate analysis identified 441 significant features (*p* ≤ 0.05; FDR ≤ 0.05); Supplementary Fig. 1b, Online Resource 2). PLS-DA based on these features showed clear separation between groups (Fig. 2a) with strong performance (Q² = 0.90; R² = 0.90; accuracy = 1.00; Fig. 2b), supported by permutation testing (*p* < 0.03; Fig. 2c).

Identification of the 441 features resulted in 190 candidate compounds: 148 assigned to confidence level 3 (tentative structure based on precursor ion [MS¹] and database/library matches; Supplementary Table 3, Online Resource 1) and 42 to level 2 (putative identification supported by MS² fragmentation and literature/database corroboration; Supplementary Table 4, Online Resource 1) [42, 49, 50].

#### Lipidomics

Data processing generated 3,028 features in positive ionization mode and 4,008 features in negative ionization mode. After merging datasets, a single matrix comprising 7,036 variables was obtained. Following pre-treatment procedures, 3,572 features were retained for chemometric analysis. PCA demonstrated good QC clustering of QC samples (Supplementary Fig. 1c, Online Resource 2), indicating analytical reproducibility.

Univariate analysis identified 69 significant features (Student’s t-test, *p* ≤ 0.05; FDR ≤ 0.05; Supplementary Fig. 1 d, Online Resource 2). PLS-DA using these features showed clear group separation (Fig. 2d). The PLS-DA model exhibited strong performance, with a predictive ability (Q²) of 0.70 and goodness of fit (R²) of 0.80 (cross-validation, Fig. 2e), and achieved perfect classification accuracy (1.00; Fig. 2e). Model robustness was confirmed by permutation test with 1,000 iterations (*p* < 0.01; Fig. 2f), indicating low probability of overfitting.

Of the among 69 significant features, 34 lipids were classified as unknown, 11 were annotated at confidence level 3 (Supplementary Table 5, Online Resource 1), and 24 at confidence level 2 (Supplementary Table 6, Online Resource 1). Identification reached species level, representing total carbon number, double bond equivalents (DBEs), and additional oxygen atoms. Annotations were confirmed by MS² fragmentation spectra using Progenesis software [42, 50]. Lipid identification were assigned following guidelines accounting for specific structural features of lipid classes [51].

#### ROC Curve Analysis

ROC analyses were performed using PLS-DA models built from identified features to assess discrimination between exposed and control groups. In metabolomics, the model showed excellent classification performance (AUC = 1.00; 95% CI: 0.99–1.00; Supplementary Fig. 2a, Online Resource 2), confirmed by high predictive accuracy in the confusion matrix (Supplementary Fig. 2b, Online Resource 2). In lipidomics, with 49% of significant features identified, the model demonstrated strong discriminatory ability (AUC = 0.94; 95% CI: 0.85–0.99; Supplementary Fig. 2c, Online Resource 2), with robust confusion matrix performance (Supplementary Fig. 2 d, Online Resource 2). These findings support an association between occupational exposure and metabolic alterations in jewelry welders.

### Spearman Correlation Analysis of Plasma Metabolites and Lipids with Toxic and Potentially Toxic Elements

**Fig. 3:**
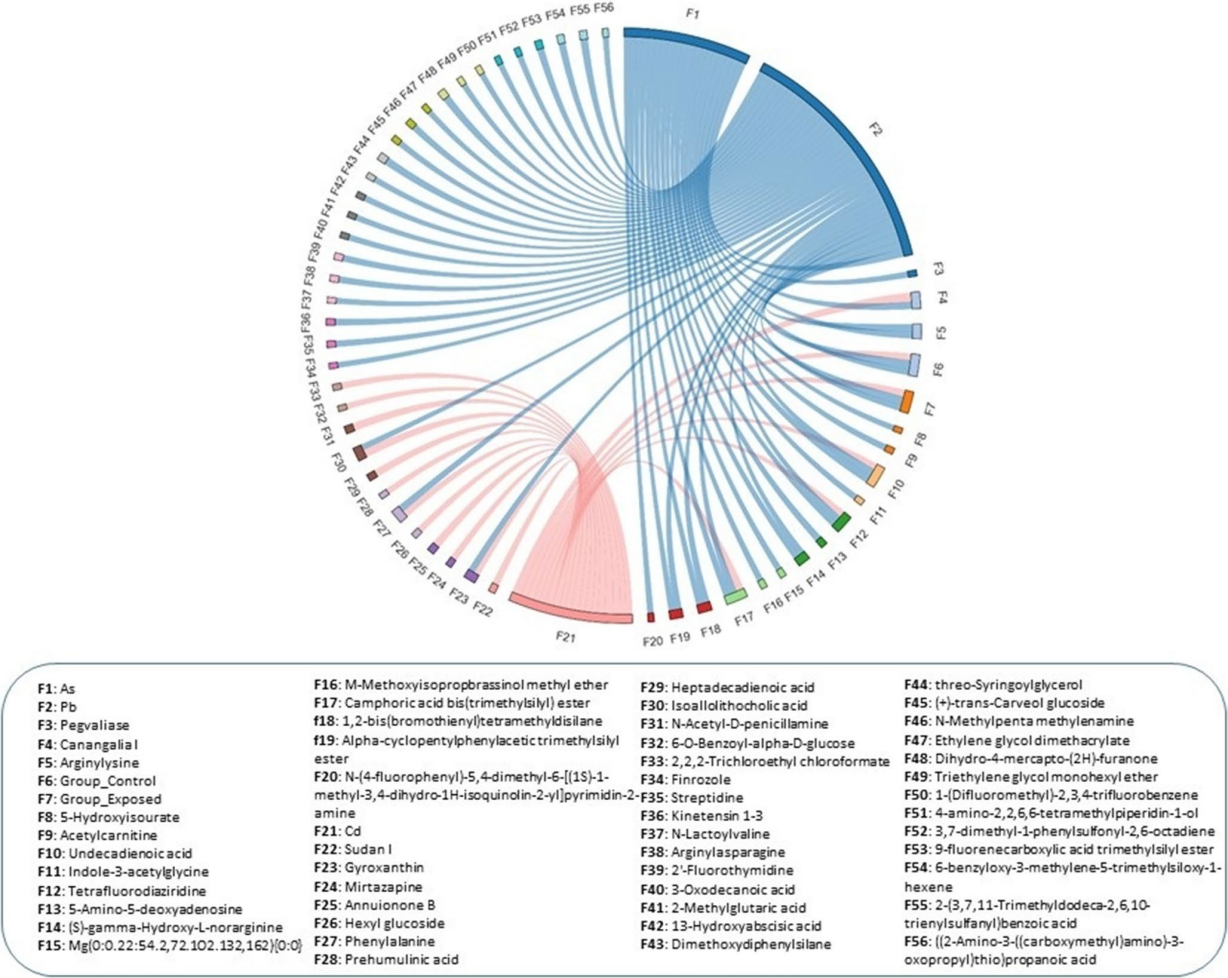
Chord diagram illustrating moderate correlations between metabolites levels and blood concentrations of PTEs, based on Spearman’s correlation coefficient (0.3 ≤ |r| ≤ 0.5). Identified metabolites and their reference codes are listed below the diagram. Each arc represents a distinct metabolite–PTE association, with arc width corresponding to the strength of correlation

From the metabolomics analysis, 53 metabolites showed significant correlations (*p* < 0.05) with at least one PTE, exhibiting moderate correlation coefficients (*r* = 0.3 to 0.5). Noteworthy examples included phenylalanine with Pb and Cd; Acetylcarnitine with As; Sudan I with Cd; Hexyl glucoside with Cd; and MG 22:5 with As. The highest number correlations were observed with Pb (24 metabolites), As (16 metabolites), and Cd (13 metabolites), as depicted in the chord diagram (Fig. 3), illustrating the broad influence of these elements on the metabolic profile.

**Fig. 4:**
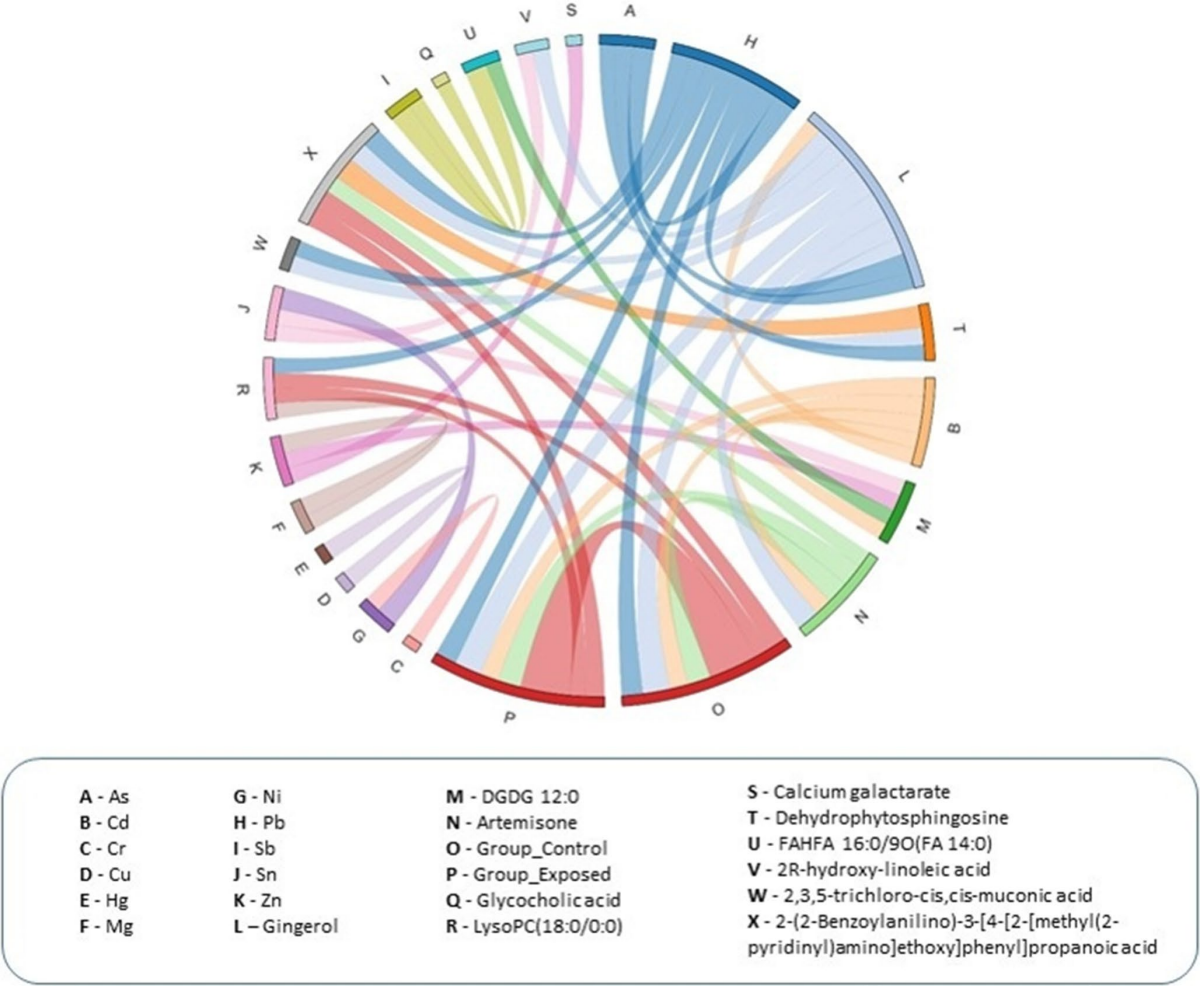
Chord diagram illustrating moderate correlations between lipids levels and blood levels of PTEs, based on Spearman’s correlation coefficient (0.3 ≤ |r| ≤ 0.5). Identified lipids and their reference codes are listed below the diagram. Each arc depicts a specific lipid–PTE association, with arc width reflecting the strength of the correlation

In the lipidomics analysis, 11 lipids presented significant correlations (*p* < 0.05) with at least one PTE, also with moderate effect sizes (*r* = 0.3 to 0.5). These included 2 lipids associated with As, 3 with Cd, 1 with Mn, 4 with Pb, 2 with Sb, 2 with Sn, and 2 with Zn. Examples included 2R-Hydroxy-linoleic acid with Sn; Gingerol with Pb, As, and Cd; Dehydrophytosphingosine with As; Glycolic acid with Sb; LysoPC 18:0 with Pb and Mn; and 2,3,5-Trichloro-cis, cis-muconic acid with Pb. These associations are visualized in the chord diagram (Fig. 4), highlighting specific lipid signatures related to PTE exposure.

### Enrichment Analysis

**Fig. 5:**
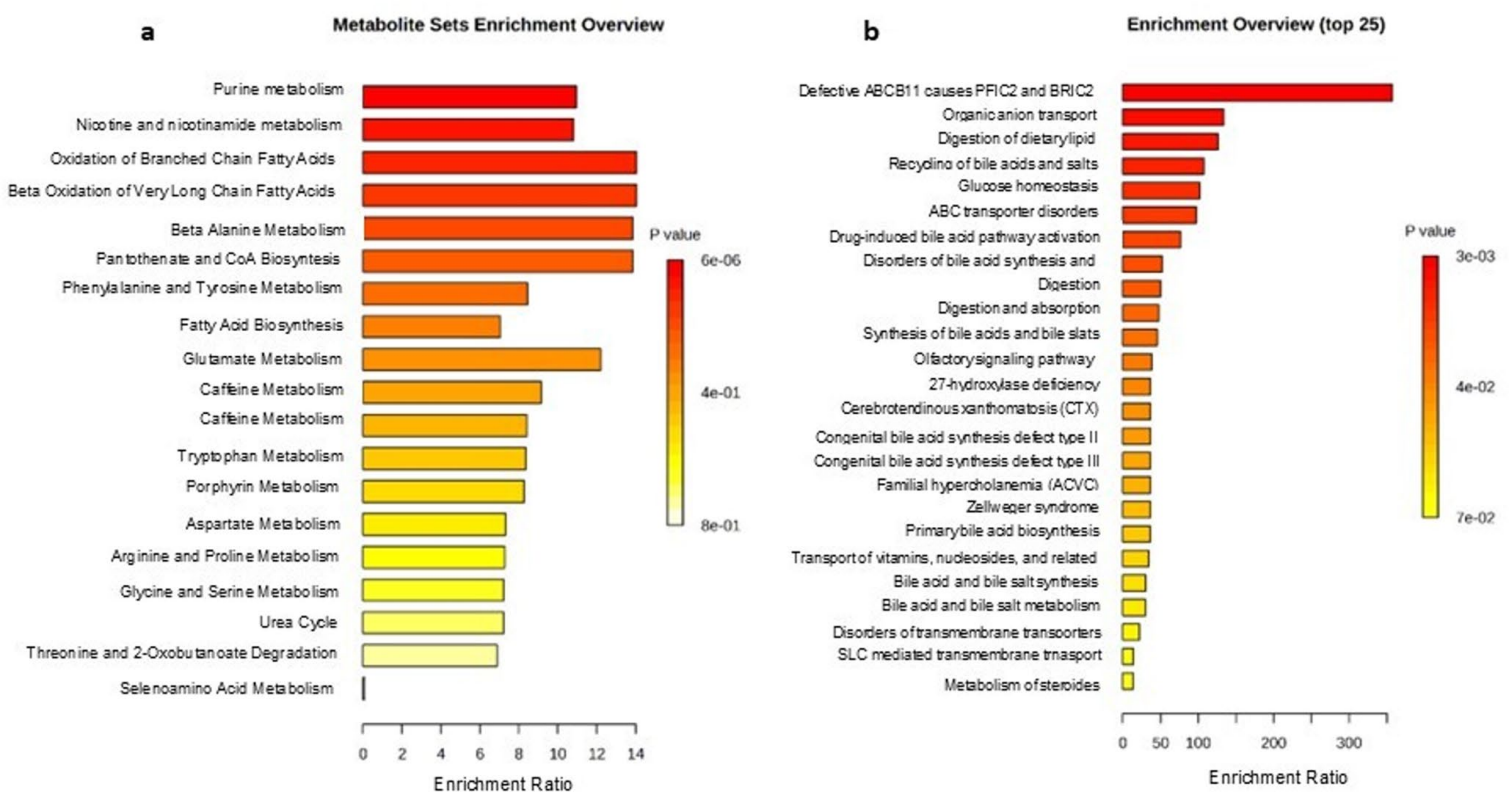
Enrichment analysis. Left panel (**a**): enrichment results for identified metabolites. Right panel (**b**): enrichment results for identified lipids. Bar charts represent the top altered biochemical pathways as determined by enrichment significance

In the metabolomics dataset, enrichment analysis revealed significant alterations in several biochemical pathways, including purine metabolism, nicotinate and nicotinamide metabolism, fatty acid oxidation, phenylalanine and tyrosine metabolism, tryptophan metabolism, and porphyrin metabolism, among others (Fig. 5a). These findings indicate direct impacts on human physiological processes and suggest systemic metabolic effects related to PTE.

For the lipidomics dataset, enrichment analysis identified potential alterations in 25 pathways, including organic anion transport, glycerolipid metabolism, digestion of dietary lipids, recycling of bile acids and salt recycling, glucose homeostasis, and processes related to ABC transporters (Fig. 5b). Notably, several of these pathways have previously been linked to PTE exposure, further supporting the biological relevance of the alterations observed.

## Discussion

To our knowledge, this study presents the first comprehensive analysis of systemic metabolic and lipidomic alterations associated with occupational exposure in informal, home-based women jewelry welders, using plasma profiles from the same samples analyzed separately for each omics layer. The results demonstrate a clear association between low blood levels of toxic and potentially toxic elements, particularly arsenic (As), cadmium (Cd), and lead (Pb), and disruptions in essential metabolic and lipid pathways. Notably, these molecular disturbances were observed at exposure levels below commonly used reference/action limits, highlighting a critical regulatory gap. Occupational agencies and regulations (e.g., OSHA and Brazil’s NR-7) establish action levels to guide workplace surveillance and intervention, whereas public health bodies (e.g., WHO and ATSDR) provide health-based guidance for environmental and public exposures. However, there remains no international consensus on truly safe internal-dose thresholds for several metals. For Pb, the 95th percentile blood Pb concentration in this cohort (4.65 µg/dL, Table 1) was ~ 12-fold below Brazil’s NR-7 limit (60 µg/dL) [46], it exceeded the Centers for Disease Control and Prevention (CDC) Blood Lead Reference Value (BLRV) (3.5 µg/dL) and approached NIOSH/ABLES threshold (≥ 5 µg/dL) [47, 48], and was close to the NIOSH/ABLES reference value for an elevated adult BLL (≥ 5 µg/dL). From a public-health perspective, the World Health Organization emphasizes that no safe blood lead has been identified [44, 45]. Similarly, blood Cd levels (GM 0.5 µg/L; P95 3.32 µg/L) were below the OSHA biological action level for medical surveillance (CdB > 5 µg/L) [43], yet were still associated with measurable molecular changes, consistent with evidence that biological effects may occur at low-level exposure.”

### Metabolomic and Lipidomic Alterations Linked to PTE Exposure

These findings indicate that, even at low blood concentrations, PTEs are associated with a distinct biochemical footprint in exposed individuals. Specifically, a subset of metabolites and lipids correlated with blood levels of As, Cd, and Pb (Fig. 3e Fig. 4, respectively), supporting exposure-related molecular perturbations.

Previous studies reported elevated Cd and Pb levels in the breathing zones of jewelers workers and in materials used in production, including jewelry products and soldering powders [17, 18]. Additionally, As and Pb were detected in household dust from the workers’ homes [11]. In the present study, these exposure profiles were accompanied by alterations consistent with disruptions in energy metabolism, amino acid metabolism, oxidative stress responses, and purine metabolism.

Among amino acids, phenylalanine was significantly correlated with Cd and Pb, suggesting perturbation of pathways linked to neurotransmitter synthesis and brain function [52]. Similar disruptions in phenylalanine-related metabolism have been reported in both environmental and occupational settings involving metal mixtures. For example, altered phenylalanine metabolism has been described in individuals living near coal refineries exposed to multiple metals, including Cd [53], and in workers occupationally exposed to As, Cd, and Pb [54]. In addition, inverse associations with other EPTs (e.g., Cu, Zn, and Hg) have been reported [55], which may further influence neurotoxic susceptibility by affecting neurotransmitter precursor availability and redox balance.

Decreased acetylcarnitine levels were observed among exposed individuals. Carnitines are essential for mitochondrial energy metabolism, facilitating the β‑oxidation of fatty acids [56]. Reduced acetylcarnitine may indicate mitochondrial dysfunction and heightened oxidative stress, given the role of carnitines in cellular protection against toxic insults [57, 58]. This mechanism is biologically relevant in metal toxicity, including, Pb exposure, which is known to promote oxidative stress and metabolic disturbances [59]. Declines in other carnitines, including 2-octenoylcarnitine and 3-hydroxyhexadecanoylcarnitine, further support a negative impact on energy metabolism. Acetylcarnitine also showed a significant correlation with As levels, and similar alterations have been reported in workers exposed to PTEs [60]. Given the neuroprotective and antioxidant properties of carnitines [61, 62], decreases in these metabolites may have implications for cognitive and neurological functions [63]. An earlier study also reported an association between C14:2 and metal exposure, particularly to Pb [64], reinforcing the consistency of carnitine-related perturbations across metal-exposed environments. While Pb levels in that cohort were lower than in the present study (Table 1), metabolomic alterations were reported in both settings, reinforcing the sensitivity of carnitine-related metabolism to Pb even at exposure levels commonly classified as low by conventional benchmarks.

Evidence from this study also points to potential occupational co-exposures. A significant correlation between Sudan I and Cd was observed. Sudan I has been reported as genotoxic and carcinogenic in experimental models [65], and its detection may reflect the use of materials related to jewelry manufacture. Together with Cd-related toxicity mechanisms (oxidative stress, mitochondrial damage, DNA damage, apoptosis) [66–68], and previous measurements of Cd in household dust from this population [11], the data highlight the value of metabolomics for detecting co-exposure signals in complex occupational environments [69, 70].

Several metabolites not directly correlated with blood PTEs concentrations, such as uric acid, aspartylphenylalanine and porphobilinogen, also emerged as markers of altered metabolism [64, 71]. Although not all of these features were significantly associated with individual PTEs, they are involved in signaling, fatty acid transport, and energy metabolism [55, 64]. Aspartylphenylalanine has been reported as a biomarker associated with multiple metals in plasma (Al, As, Ba, and Zn) [64].

Uric acid functions as an important plasma antioxidant, and both elevated (hyperuricemia) and reduced (hypouricemia) levels have clinical significance [72–74]. While hyperuricemia has been reported in Pb-exposed workers [71, 75, 76] and National Health and Nutrition Examination Survey (NHANES, 2007–2016) data suggest that metal mixture exposures may increase uric acid [76],, reduced uric acid was observed in exposed workers. These findings align with Korea National Health and Nutrition Examination Survey (KNHANES) reports of inverse associations between blood cadmium and uric acid, particularly among women [77]. In addition, the elevated blood Cd observed in exposed women is consistent with previous findings of increased urinary Cd concentration in this population [18]. Metal exposure has been linked to disruption in purine metabolism [78, 79], and decreases in xanthylic acid (a uric acid precursor) [80, 81], may suggest interference with in purine catabolism [82, 83].

Broader studies such as NHANES [76] offer valuable population insights; however, the exposure profiles here differ and may produce unique health effects not seen in larger surveys. Reduced uric acid in jewelry welders suggests early renal dysfunction or depletion of antioxidant defenses from chronic metal exposure. The role of uric acid as a biomarker remains debated. These results underscore the need for focused studies on vulnerable groups, notably informal female workers facing complex exposures.

Lipidomic analysis identified 69 significantly altered lipids features, of which 35 were annotated at higher confidence. The altered lipid profile supports links between PTE exposure and lipid metabolism in this occupational environment. Identified lipids included fatty acid–related species (e.g., 2R-hydroxy-linoleic acid), phospholipids (e.g., LysoPC(18:0)); and bioactive exogenous metabolites (e.g., gingerol). Among these, 11 lipids were significantly correlated with blood PTEs, including As, Cd, Pb, Sb, Mn, and Zn. Lysophosphatidylcholines (LysoPCs), are important for lipid metabolism and membrane integrity [84]. The observed correlation of LysoPC(18:0) with Mn and Pb suggests potential interactions between metal exposure and lipid homeostasis that remain underexplored. Prior work has shown that chronic As exposure induces dose-dependent changes in LysoPC species [84], consistent with the notion that PTEs may disrupt lipid homeostasis and inflammatory signaling even at relatively low concentrations.

### Biological Pathways and Mechanisms of Toxicity

Pathway enrichment analysis indicated alterations in purine metabolism, nicotinate and nicotinamide metabolism, fatty acid oxidation, phenylalanine and tyrosine metabolism, tryptophan metabolism and porphyrin metabolism. These pathways have been linked to reduced kidney dysfunction and the development of kidney disease or acute kidney injury in populations exposed to metals [85–87]. These metabolic changes also involve phenylalanine hydroxylase activity, which is attenuated under inflammatory conditions [64]. Disruption of purine metabolism supports the concept that PTEs may contribute to oxidative stress, a recognized factor in chronic diseases [55, 88].

Within amino acid metabolism, tryptophan and tyrosine pathways, critical for the neurotransmitter synthesis (e.g., serotonin and dopamine), were dysregulated in association with Cd and Pb exposure [53, 55, 89, 90]. Such alterations have been associated with oxidative stress and neuropsychological outcomes, including mood and cognitive disturbances, symptoms such as headaches and sleep disturbances have been reported among metal-exposed workers [33]. Occupational exposure to PTEs has also been linked to changes in human gene expression even at relatively low exposure levels [91], supporting the hypothesis that these elements can interfere with key biological pathways. In addition, a previous study has documented that children and adolescents involved in this productive arrangement tend to sleep less and have restricted opportunities for leisure and study time [92].

A reduction in porphobilinogen observed among exposed workers is consistent with the inhibition of ALAD, a recognized effect of lead toxicity. These findings underscore the toxicological relevance of such exposures and align with previous evidence that even low-level Pb exposure is associated with biochemical and neurological effects (e.g., hypertension, neuropathy, memory loss, irritability, and headaches) [33, 93], highlighting the sensitivity of heme biosynthesis pathways to modest lead burdens.

Complementary lipidomics enrichment analysis revealed significant changes in ABC transporter activity, glycerolipid metabolism, and glucose homeostasis, among others. Pb exposure has been linked to disruptions in ABC transporter pathways, potentially contributing to adverse health outcomes including metabolic diseases and cancer [94]. Previous research by our group also found significantly higher Pb concentrations in exposed individuals [78], a result that was replicated in this study (*p* < 0.001).

Alterations in glycerolipid metabolism associated with Pb exposure may influence inflammation and immune responses [66, 95, 96]. In addition, Cd, a known hyperglycemic metal promotes increased blood glucose and dysregulation of glucose homeostasis [97]. In accordance with these findings, significant differences in blood glucose (*p* = 0.03) and Cd levels (*p* < 0.001) were observed in this population, with blood Cd levels positively correlated with elevated glucose [33].

Glycerolipid metabolism, another altered pathway identified, is fundamental for human biochemistry and is disrupted by As exposure, contributing to oxidative stress [98, 99]. In the current study, blood As concentrations were significantly higher in welders than controls (*p* = 0.01). Arsenic exposure has been shown to perturb glycerolipid metabolism, potentially increasing cardiovascular disease risk [100–103].

Major strengths of this work include parallel metabolomics and lipidomics profiling of plasma samples from women jewelry welders in informal home-based settings, correlated with previously reported blood PTE concentrations (As, Cd, Pb). This multi-omics approach using the same biological samples elucidates molecular mechanisms of low-level chronic exposure effects in a vulnerable population, advancing exposome research in occupational public health.

### Study Limitations

It is important to highlight that this was a cross-sectional study; therefore, certain limitations must be considered. First, although recruitment has been conducted with support from ACS linked to the ESF and has included all families identified by the local teams, the use of non-probabilistic recruitment strategies may introduce selection bias and limit the external validity of the results. Recruitment was conducted across different neighborhoods, where informal welders were registered by the ESF program, with standardized inclusion criteria applied to both exposure groups. The sample also included participants with diverse socioeconomic profiles. Additionally, previous data from study by Ferreira et al. [17] guided participant identification and improved understanding of the studied population and work processes. Therefore, the results should be interpreted cautiously regarding external validity, being most directly applicable to populations with similar socio-occupational contexts and recruitment strategies.

The modest sample size limits statistical power to detect smaller magnitude effects in metabolomics and lipidomics analyses. No formal a priori power analysis was conducted due to the exploratory design and use of a convenience sample. Although rigorous quality control procedures, FDR correction, and model validation (cross-validation and permutation testing) were applied, results should be considered exploratory and confirmed in larger, independent cohorts.

A substantial proportion of metabolite annotations remained at MSI level 3 (putative identification); therefore, these signals should be interpreted as hypothesis-generating rather than definitive identifications.

Finally, as the study was conducted in a single municipality in the state of São Paulo, generalizability to other regions and occupational contexts is limited. Differences in work processes, environmental conditions, and exposure profiles may modify the magnitude of the observed associations. Nevertheless, comparable contexts of occupational exposure in informal work have been described elsewhere, including informal e-waste recyclers in Ghana [104], informal foundry workers in South Africa [105], occupational particulate matter exposure informal work settings [106], and toxic metal exposure among informal landfill workers [107], both in Indonesia. Thus, while context-specific, our findings may inform hypotheses for similar informal occupational environments. Replication in larger, geographically diverse cohorts is needed before broader inference or clinical translation.

## Conclusions

This study demonstrates subtle yet significant biochemical changes induced by chronic occupational exposure to toxic and potentially toxic elements (PTEs), even at low concentrations. The results provide new insights into health effects of informal occupational activities, underscoring their impact on exposed workers. Omics analyses revealed disruptions in pathways related to purine, porphyrin, and glycerolipid metabolism, highlighting the biological relevance of exposures often deemed safe by regulatory standards. These findings emphasize health risks posed by informal work environment and stress the importance of biomarker-based monitoring to protect vulnerable populations.

## Supplementary Information

Below is the link to the electronic supplementary material.

Supplementary Material 1

## Data Availability

The metabolomics data (.raw and processed files) generated and analyzed in this study will be deposited in a public data repository (e.g., MetaboLights) after acceptance of this manuscript. Until then, the data are available from the corresponding author on reasonable request.
