# Supplementary material for: Low-level Blood Multiple Metals Exposure from Informal Jewelry Welding Alters Metabolomic and Lipidomic Profiles in Brazilian Women
Source: Biol Trace Elem Res. 2026 Apr 16;204(8):6121–37. doi: 10.1007/s12011-026-05096-4 (PMC13369782; doi:10.1007/s12011-026-05096-4)
Supplement: Supplementary file 1 — Supplementary Material 1 [file 12011_2026_5096_MOESM1_ESM.docx]

**Low-level blood multiple metals exposure from informal welding activities alters metabolomic and lipidomic profiles in Brazilian women**

Alda Neis Miranda de Araújo^a,b,c^* (<https://orcid.org/0000-0003-3649-8683>), Vinicius Guimarães Ferreira^b,c^ (<https://orcid.org/0000-0001-8053-3867>), Isabelle Nogueira Leroux^b,c^ (<https://orcid.org/0000-0003-1475-2281>), Danilo Cardoso de Oliveira^d^ (<https://orcid.org/0000-0003-3649-8683>), Andreia M Porcari^d^ (<https://orcid.org/0000-0003-4244-8594>), Kelly Polido Kaneshiro Olympio^b,c^**^†^*** (<https://orcid.org/0000-0002-4257-8295>), Nilson Antônio de Assunção^a,e^**^†^** (<https://orcid.org/0000-0002-3747-0415>).

*^a^ Graduate Program in Translational Medicine, Paulista School of Medicine, Department of Medicine, Federal University of São Paulo, São Paulo, Brazil.*

*^b^ Departament of Environmental Health, School of Public Health, University of São Paulo, Brazil.*

*^c^ The Human Exposome Research Group/Expossoma e Saúde do Trabalhador – eXsat, School of Public Health, University of Sao Paulo, São Paulo, Brazil.*

*^d^ MS4Life Laboratory of Mass Spectrometry, Health Sciences graduate Program, São Francisco University, Bragança Paulista, Brazil.*

*^e^ Department of Chemistry, Institute of Environmental, Chemical and Pharmaceutical Sciences Federal University of São Paulo, Diadema, São Paulo, Brazil.*

**^†^**These authors have contributed equally to this work and share last authorship

***Corresponding authors:**

E-mail: [aldaaraujo@usp.br](mailto:aldaaraujo@usp.br); [kellypko@usp.br](mailto:kellypko@usp.br).

**Online Resource 1 – Supplementary tables**

**Supplementary Table 1.** Separation gradient for the metabolomics analysis by UHPLC-MS.

| Time (min) | % Solvent A | % Solvent B | Flow rate (mL/min) |
| --- | --- | --- | --- |
| 0.00 | 99 | 1 | 0.400 |
| 3.00 | 98 | 2 | 0.400 |
| 10.00 | 70 | 30 | 0.400 |
| 15.00 | 50 | 50 | 0.400 |
| 18.00 | 20 | 80 | 0.400 |
| 20.00 | 10 | 90 | 0.400 |
| 22.00 | 5 | 95 | 0.400 |
| 26.01 | 1 | 99 | 0.400 |
| 28.01 | 1 | 99 | 0.400 |
| 30.00 | 99 | 1 | 0.400 |

**Supplementary** **Table 2**: Separation gradient for the lipidomic analysis by UHPLC-MS.

| Time (min) | % Solvent A | % Solvent B | Flow rate (mL/min) |
| --- | --- | --- | --- |
| 0.00 | 99 | 1 | 0.400 |
| 3.00 | 97 | 3 | 0.400 |
| 6.00 | 94 | 6 | 0.400 |
| 1.00 | 60 | 40 | 0.400 |
| 1.00 | 30 | 70 | 0.400 |
| 1.00 | 10 | 90 | 0.400 |
| 1.00 | 1 | 99 | 0.400 |
| 20.00 | 1 | 99 | 0.400 |
| 20.30 | 99 | 1 | 0.400 |
| 23.00 | 99 | 1.0 | 0.400 |

Supplementary Table 3. Metabolites identified at the confidence level 3.

| **Feature** | **Identified lipids** | **HMDB** | **Acquisition** | **Adduct** | **Formula** | **Identification level** |
| --- | --- | --- | --- | --- | --- | --- |
| 6.21_256.9964*m/z* | Alpha-(3-phenoxyphenyl)ethyl bromide | JP004077 | - | M-H2O-H | C₁₄H₁₃BrO | 3 |
| 0.74_691.3789*m/z* | Gyroxanthin | LMPR01070747 | - | M+Cl | C₄₂H₅₆O₆ | 3 |
| 13.24_215.1641*m/z* | Monohexyl triethylene glycol ether | HMDB0259194 | - | M-H2O-H | C₁₂H₂₆O₄ | 3 |
| 5.46_181.0084 *m/z* | 1-(Difluoromethyl)-2,3,4-trifluorobenzene | HMDB0243802 | - | M-H | C₇H₃F₅ | 3 |
| 8.29_344.1830n*m/z* | Bis(trimethylsilyl) camphoric acid ester | JP001046 | - | M+Cl, M+FA-H | C₁₆H₃₂O₄Si₂ | 3 |
| 11.63_445.2408*m/z* | Fluocortin butyl | HMDB0252350 | - | M-H | C₂₆H₃₅FO₅ | 3 |
| 2.00_172.0343n | 2,6-Difluorophenylacetic acid | JP011241 | - | M-H, M+FA-H | C₈H₆F₂O₂ | 3 |
| 0.85_260.0268*m/z* | Tiamenidine | HMDB0259067 | - | M+FA-H | C₈H₁₀ClN₃S | 3 |
| 1.33_180.9895*m/z* | Sevoflurane | HMDB0015366 | - | M-H2O-H | C₄H₃F₇O | 3 |
| 17.32_293.1743*m/z* | Artemether | HMDB0242731 | - | M-H2O-H | C₁₇H₂₈O₅ | 3 |
| 7.03_331.1353*m/z* | Dienestrol diacetate | HMDB0251232 | - | M-H2O-H | C₂₂H₂₂O₄ | 3 |
| 10.44_213.0759*m/z* | Glycerol 1-propanoate, diacetate | HMDB0031640 | - | M-H2O-H | C₁₀H₁₆O₆ | 3 |
| 8.86_403.1951*m/z* | Cinnarizine | HMDB0014708 | - | M+Cl | C₂₆H₂₈N₂ | 3 |
| 13.95_301.1998*m/z* | Arginyl-lysine | HMDB0028714 | - | M-H | C₁₂H₂₆N₆O₃ | 3 |
| 10.91_371.1687*m/z* | Meclizine | HMDB0014875 | - | M-H2O-H | C₂₅H₂₇ClN₂ | 3 |
| 29.01_456.2349*m/z* | LysoPC 10:0 (Lysophosphatidylcholine 10:0) | HMDB0003752 | - | M+FA-H | C₁₈H₃₈NO₇P | *species level* |
| 5.47_328.9533*m/z* | Tris(2-chloroethyl) phosphate | HMDB0259276 | - | M+FA-H | C₆H₁₂Cl₃O₄P | 3 |
| 0.74_997.3220*m/z* | Curcumin 4',4''-O-beta-D-digentioside | HMDB0304299 | - | M-H2O-H | C₄₅H₆₀O₂₆ | 3 |
| 12.17_241.1069*m/z* | Tripropanoate of glycerol | HMDB0032857 | - | M-H2O-H | C₁₂H₂₀O₆ | 3 |
| 16.52_459.2001*m/z* | Prednisolone hemisuccinate | HMDB0245744 | - | M-H | C₂₅H₃₂O₈ | 3 |
| 13.07_275.0680*m/z* | 3-(3,4,5-Trimethoxyphenyl)propanoic acid | HMDB0030254 | - | M+Cl | C₁₂H₁₆O₅ | 3 |
| 9.29_423.1774*m/z* | PG(6:0/6:0) | LMGP04010030 | - | M-H2O-H | C₁₈H₃₅O₁₀P | *species level* |

Supplementary Table 3. Metabolites identified at the confidence level 3 (continued).

| **Feature** | **Identified lipids** | **HMDB** | **Acquisition** | **Adduct** | **Formula** | **Identification level** |
| --- | --- | --- | --- | --- | --- | --- |
| 10.56_261.0869*m/z* | Aspartylphenylalanine | HMDB0000706 | - | M-H2O-H | C₁₃H₁₆N₂O₅ | 3 |
| 11.28_325.1650*m/z* | Prehumulone acid | HMDB0030148 | - | M+FA-H | C₁₆H₂₄O₄ | 3 |
| 17.23_460.2686*m/z* | Neoline | HMDB0242684 | + | M+Na | C₂₄H₃₉NO₆ | 3 |
| 17.23_434.2431*m/z* | Sphingosine 1-phosphate (d19:1-P) | HMDB0060062 | + | M+K | C₁₉H₄₂NO₅P | *species level* |
| 21.37_686.4591*m/z* | HexCer-NS d32:5 | LipidBlast041692 | + | M+Na | C₃₈H₆₅NO₈ | *species level* |
| 0.72_669.3705*m/z* | DGDG 16:0 | LipidBlast033076 | + | M+H | C₃₁H₅₆O₁₅ | *species level* |
| 9.26_389.1790*m/z* | β-D-Glucopyranosyl-11-hydroxyjasmonic acid | HMDB0039964 | + | M+H | C₁₈H₂₈O₉ | 3 |
| 13.95_223.1322*m/z* | Annuionone B | HMDB0032121 | + | M+H | C₁₃H₁₈O₃ | 3 |
| 11.06_158.1173*m/z* | (S)-Homostachydrine | HMDB0033433 | + | M+H | C₈H₁₅NO₂ | 3 |
| 10.12_174.0575*m/z* | N-Acetyl-L-methionine | HMDB0011745 | + | M+H-H2O | C₇H₁₃NO₃S | 3 |
| 8.85_311.1124*m/z* | Nivalenol | HMDB0004304 | - | M-H | C₁₅H₂₀O₇ | 3 |
| 14.57_357.2267*m/z* | Kinetensin 1-3 | HMDB0012983 | - | M-H | C₁₅H₃₀N₆O₄ | 3 |
| 10.99_343.1016*m/z* | Clotrimazole | HMDB0001922 | - | M-H | C₂₂H₁₇ClN₂ | 3 |
| 8.06_333.1901*m/z* | Arginine ornithine | HMDB0248583 | - | M+FA-H | C₁₁H₂₄N₆O₃ | 3 |
| 8.07_359.1697*m/z* | Perilloside A | HMDB0038706 | - | M+FA-H | C₁₆H₂₆O₆ | 3 |
| 10.55_229.0608*m/z* | 5-Hydroxyindoleacetylglycine | HMDB0004185 | - | M-H2O-H | C₁₂H₁₂N₂O₄ | 3 |
| 11.94_283.1538*m/z* | Arginylglutamine | HMDB0028707 | - | M-H2O-H | C₁₁H₂₂N₆O₄ | 3 |
| 9.83_347.1692*m/z* | Arginylglutamine | HMDB0028707 | - | M+FA-H | C₁₁H₂₂N₆O₄ | 3 |
| 11.46_327.1068*m/z* | Trimethylsilyl 9-fluorenecarboxylic acid ester | JP001088 | - | M+FA-H | C₁₇H₁₈O₂Si | 3 |
| 8.08_213.0750*m/z* | Arginine | HMDB0000517 | + | M+K | C₆H₁₄N₄O₂ | 3 |
| 12.33_387.2006*m/z* | Dihydroroseoside | HMDB0040614 | - | M-H | C₁₉H₃₂O₈ | 3 |
| 10.03_197.0563*m/z* | N1-Methyl-2-pyridone-5-carboxamide | HMDB0004193 | - | M+FA-H | C₇H₈N₂O₂ | 3 |
| 9.66_197.1174*m/z* | Undecanoic acid | HMDB0000888 | - | M-H2O-H | C₁₁H₂₀O₄ | *Species level* |

Supplementary Table 3. Metabolites identified at the confidence level 3 (continued).

| **Feature** | **Identified lipids** | **HMDB** | **Acquisition** | **Adduct** | **Formula** | **Identification level** |
| --- | --- | --- | --- | --- | --- | --- |
| 8.06_333.1901*m/z* | Arginine ornithine | HMDB0248583 | - | M+FA-H | C₁₁H₂₄N₆O₃ | 3 |
| 8.07_359.1697*m/z* | Perilloside A | HMDB0038706 | - | M+FA-H | C₁₆H₂₆O₆ | 3 |
| 10.55_229.0608*m/z* | 5-Hydroxyindoleacetylglycine | HMDB0004185 | - | M-H2O-H | C₁₂H₁₂N₂O₄ | 3 |
| 11.94_283.1538*m/z* | Arginylglutamine | HMDB0028707 | - | M-H2O-H | C₁₁H₂₂N₆O₄ | 3 |
| 9.05_288.1514*m/z* | Zolpidem | HMDB0005023 | - | M-H2O-H | C₁₉H₂₁N₃O | 3 |
| 23.39_254.8787*m/z* | 2,2,2-Trichloroethyl chloroformate | HMDB0245336 | - | M+FA-H | C₃H₂Cl₄O₂ | 3 |
| 10.66_259.1170*m/z* | 3,7-Dimethyl-1-phenylsulfonyl-2,6-octadiene | JP009933 | - | M-H2O-H | C₁₆H₂₂O₂S | 3 |
| 9.55_267.0859*m/z* | Acetopine | HMDB0039111 | - | M+Cl | C₈H₁₆N₄O₄ | 3 |
| 7.88_257.1383*m/z* | Daumone | HMDB0250877 | - | M-H2O-H | C₁₃H₂₄O₆ | 3 |
| 9.43_387.2003*m/z* | 7α-Thiomethylspironolactone | HMDB0246238 | - | M-H | C₂₃H₃₂O₃S | 3 |
| 7.15_233.1018*m/z* | 2,3-Butanediol glycoside | HMDB0040822 | - | M-H2O-H | C₁₀H₂₀O₇ | 3 |
| 10.87_369.1530*m/z* | Galactosylhydroxylysine | HMDB0000600 | - | M+FA-H | C₁₂H₂₄N₂O₈ | 3 |
| 2.76_157.0245*m/z* | Trimethylsilyl-1,3-butadiyne | JP011939 | - | M+Cl | C₇H₁₀Si | 3 |
| 9.20_275.1128*m/z* | Triethyl citrate | HMDB0034263 | - | M-H | C₁₂H₂₀O₇ | 3 |
| 28.01_318.2142n | Pegvaliase | HMDB0256204 | + | M+H-H2O, M+H, M+Na | C₁₅H₃₀N₂O₅ | 3 |
| 7.33_299.1134*m/z* | Histidyltyrosine | HMDB0028897 | - | M-H2O-H | C₁₅H₁₈N₄O₄ | 3 |
| 8.45_333.1537*m/z* | Arginylasparagine | HMDB0028704 | - | M+FA-H | C₁₀H₂₀N₆O₄ | 3 |
| 9.13_239.0913*m/z* | (+)-gamma-Hydroxy-L-homoarginine | HMDB0301838 | - | M+Cl | C₇H₁₆N₄O₃ | 3 |
| 8.65_206.0810*m/z* | Moroxydine | HMDB0254889 | - | M+Cl | C₆H₁₃N₅O | 3 |
| 5.22_236.0590*m/z* | N-Acetyl-dl-penicillamine | HMDB0255055 | - | M+FA-H | C₇H₁₃NO₃S | 3 |
| 8.13_165.0787n | Phenylalanine | HMDB0000159 | + | M+H-H2O, M+H | C₉H₁₁NO₂ | 3 |
| 10.63_289.0911*m/z* | Dimethoxydiphenylsilane | JP008885 | - | M+FA-H | C₁₄H₁₆O₂Si | 3 |

Supplementary Table 3. Metabolites identified at the confidence level 3 (continued).

| **Feature** | **Identified lipids** | **HMDB** | **Acquisition** | **Adduct** | **Formula** | **Identification level** |
| --- | --- | --- | --- | --- | --- | --- |
| 7.55_283.0806*m/z* | N7-(2-Carbamoyl-2-hydroxyethyl)guanine | HMDB0255307 | - | M+FA-H | C₈H₁₀N₆O₃ | 3 |
| 0.85_226.9755*m/z* | Trimellitic anhydride | HMDB0259233 | - | M+Cl | C₉H₄O₅ | 3 |
| 23.24_254.8785*m/z* | 2,2,2-Trichloroethyl chloroformate | HMDB0245336 | - | M+FA-H | C₃H₂Cl₄O₂ | 3 |
| 7.34_317.1592*m/z* | S-Japonin | HMDB0035802 | - | M-H2O-H | C₁₉H₂₈O₃S | 3 |
| 9.61_201.1113*m/z* | 1,3,4-Trimethyl-1-phenyl-1-silacyclopent-3-ene | JP011688 | - | M-H | C₁₃H₁₈Si | 3 |
| 6.97_227.0232*m/z* | 2,3,5,6-Tetramethylpyrazine phosphate | HMDB0245439 | - | M-H | C₈H₉N₂O₄P | 3 |
| 10.57_419.2259*m/z* | UR-12670 | HMDB0259709 | - | M-H2O-H | C₂₈H₃₀N₄O | 3 |
| 6.91_273.0281*m/z* | (3-Nitrobenzoyl)alanine | HMDB0243652 | - | M+Cl | C₁₀H₁₀N₂O₅ | 3 |
| 7.39_139.0393*m/z* | Succinylacetone | HMDB0000635 | - | M-H2O-H | C₇H₁₀O₄ | 3 |
| 8.94_225.0756*m/z* | N(omega)-Hydroxyarginine | HMDB0004224 | - | M+Cl | C₆H₁₄N₄O₃ | 3 |
| 25.70_163.0072*m/z* | Dihydro-4-mercapto-3(2H)-furanone | HMDB0039786 | - | M+FA-H | C₄H₆O₂S | 3 |
| 8.55_231.1218*m/z* | Trimethylsilyl ester of 4-butylbenzoic acid | JP001910 | - | M-H2O-H | C₁₄H₂₂O₂Si | 3 |
| 9.81_301.1636*m/z* | Arginylglutamine | HMDB0028707 | - | M-H | C₁₁H₂₂N₆O₄ | 3 |
| 1.21_357.0294*m/z* | Trisodium edetate | HMDB0301767 | - | M-H | C₁₀H₁₃N₂Na₃O₈ | 3 |
| 7.76_283.0811*m/z* | 6-O-Benzoyl-alpha-D-glucose | HMDB0303039 | - | M-H | C₁₃H₁₆O₇ | 3 |
| 9.60_154.0627n | Hydroxytyrosol | HMDB0005784 | - | M-H2O-H, M-H | C₈H₁₀O₃ | 3 |
| 7.95_245.1381*m/z* | Hexyl glycoside | HMDB0031688 | - | M-H2O-H | C₁₂H₂₄O₆ | *Species level* |
| 9.02_343.1739*m/z* | N-(4-Fluorophenyl)-4,5-dimethyl-6-[(1S)-1-methyl-3,4-dihydro-1H-isoquinolin-2-yl]pyrimidin-2-amine | HMDB0244857 | - | M-H2O-H | C₂₂H₂₃FN₄ | 3 |
| 19.61_213.0756*m/z* | Arginine | HMDB0000517 | + | M+K | C₆H₁₄N₄O₂ | 3 |
| 0.74_894.8394*m/z* | Cer-NS d57:1: Ceramide-NS d57:1 | LipidBlast386487 | - | M+Cl | C₅₇H₁₁₃NO₃ | *Species level* |
| 0.85_357.0296*m/z* | Doxantrazole | HMDB0251600 | - | M+FA-H | C₁₄H₈N₄O₃S | 3 |

Supplementary Table 3. Metabolites identified at the confidence level 3 (continued).

| **Feature** | **Identified lipids** | **HMDB** | **Acquisition** | **Adduct** | **Formula** | **Identification level** |
| --- | --- | --- | --- | --- | --- | --- |
| 7.74_359.1702*m/z* | (+)-trans-Carveol glycoside | HMDB0029849 | - | M+FA-H | C₁₆H₂₆O₆ | 3 |
| 8.33_349.1433*m/z* | Resiquimod | HMDB0257161 | - | M+Cl | C₁₇H₂₂N₄O₂ | 3 |
| 17.05_449.1712*m/z* | LPG 11:0 | LipidBlast454428 | - | M+Cl | C₁₇H₃₅O₉P | *Species level* |
| 12.86_371.1709*m/z* | Pyrrolutamyl-phenylalanyl-prolinamide | HMDB0256991 | - | M-H | C₁₉H₂₄N₄O₄ | 3 |
| 12.25_359.1692*m/z* | 3-Ethoxy-6-(1,5-dihydroxy-5-methyl-4-phenylthio-hexyl)-2-cyclohexen-1-one | JP004598 | - | M-H2O-H | C₂₁H₃₀O₄S | 3 |
| 8.02_389.1797*m/z* | p-Fluorofentanyl | HMDB0256037 | - | M+Cl | C₂₂H₂₇FN₂O | 3 |
| 8.47_389.1799*m/z* | p-Fluorofentanyl | HMDB0256037 | - | M+Cl | C₂₂H₂₇FN₂O | 3 |
| 6.09_291.1434*m/z* | 3-Methylene-5-phenyl-5-trimethylsiloxy-1-pentene | JP002381 | - | M+FA-H | C₁₅H₂₂OSi | 3 |
| 10.40_183.1017*m/z* | Sebacic acid | HMDB0000792 | - | M-H2O-H | C₁₀H₁₈O₄ | 3 |
| 11.83_259.0730*m/z* | 2'-Fluorothymidine | HMDB0245532 | - | M-H | C₁₀H₁₃FN₂O₅ | 3 |
| 10.73_258.1045*m/z* | Coelenteramine | MoNA011510 | - | M-H2O-H | C₁₇H₁₅N₃O | 3 |
| 29.01_148.9381*m/z* | Selenocysteine | HMDB0003288 | - | M-H2O-H | C₃H₆NO₂Se | 3 |
| 8.96_377.0656*m/z* | Glucocaffeic acid | HMDB0034313 | - | M+Cl | C₁₅H₁₈O₉ | 3 |
| 8.74_203.1280*m/z* | Tetraglyme | HMDB0258894 | - | M-H2O-H | C₁₀H₂₂O₅ | 3 |
| 0.95_200.9207*m/z* | 4-Iodophenol | HMDB0246477 | - | M-H2O-H | C₆H₅IO | 3 |
| 9.85_463.1803*m/z* | Lofepramine | HMDB0254150 | - | M+FA-H | C₂₆H₂₇ClN₂O | 3 |
| 19.12_304.1575*m/z* | Prolinamide | OUF00321 | - | M-H | C₁₈H₁₉N₅ | 3 |
| 11.47_243.1587*m/z* | 4-Methoxy-2-methyl-3-trimethylsilylmethyl-3-phenyl-1-butene | JP010950 | - | M-H2O-H | C₁₆H₂₆OSi | 3 |
| 9.86_245.1379*m/z* | 3-Methylene-5-phenyl-5-trimethylsiloxy-1-pentene | JP002381 | - | M-H | C₁₅H₂₂OSi | 3 |
| 6.75_283.0913*m/z* | 2,4-Diamino-5,6-dihydroxypyrimidine | HMDB0303378 | - | M-H | C₈H₁₂N₈O₄ | 3 |

Supplementary Table 3. Metabolites identified at the confidence level 3 (continued).

| **Feature** | **Identified lipids** | **HMDB** | **Acquisition** | **Adduct** | **Formula** | **Identification level** |
| --- | --- | --- | --- | --- | --- | --- |
| 10.06_289.1635*m/z* | 6-Benzoyloxy-3-methylene-5-trimethylsiloxy-1-hexene | JP002384 | - | M-H | C₁₇H₂₆O₂Si | 3 |
| 6.58_275.1119*m/z* | 1-(Trimethylsiloxy)-1,2-dihydro-1,2-methanonaphthalene | JP002419 | - | M+FA-H | C₁₄H₁₈OSi | 3 |
| 7.86_307.1368*m/z* | Streptidine | HMDB0258506 | - | M+FA-H | C₈H₁₈N₆O₄ | 3 |
| 8.34_277.1276*m/z* | 3,7-Dimethyl-1-phenylsulfonyl-2,6-octadiene | JP009933 | - | M-H | C₁₆H₂₂O₂S | 3 |
| 23.75_286.8405*m/z* | Pentachlorophenyl acetate | JP007528 | - | M-H2O-H | C₈H₃Cl₅O₂ | 3 |
| 7.39_291.1430*m/z* | 4-(Pentamethyldisilanyl)phenyltrimethylsilylmethanol | JP001957 | - | M-H2O-H | C₁₅H₃₀OSi₃ | 3 |
| 0.75_1047.4701*m/z* | PIP(6-keto-PGF1alpha/16:1(9Z)) | HMDB0278548 | - | M+FA-H | C₄₅H₈₀O₂₀P₂ | *Species level* |
| 8.78_163.0393*m/z* | Phenylpyruvic acid | HMDB0000205 | - | M-H | C₉H₈O₃ | 3 |
| 8.74_361.1840*m/z* | N-(4-Fluorophenyl)-4,5-dimethyl-6-[(1S)-1-methyl-3,4-dihydro-1H-isoquinolin-2-yl]pyrimidin-2-amine | HMDB0244857 | - | M-H | C₂₂H₂₃FN₄ | 3 |
| 8.21_125.0236*m/z* | Carbohydrazide | HMDB0249636 | - | M+Cl | CH₆N₄O | 3 |
| 0.78_212.0197*m/z* | Quinolinic acid | HMDB0000232 | - | M+FA-H | C₇H₅NO₄ | 3 |
| 13.98_419.1907*m/z* | 6-Epi-7-isocucurbic acid glycoside | HMDB0029782 | - | M+FA-H | C₁₈H₃₀O₈ | 3 |
| 10.63_243.0874*m/z* | threo-Syringylglycerol | HMDB0031237 | - | M-H | C₁₁H₁₆O₆ | 3 |
| 9.21_267.0848*m/z* | 9-(2-Aminopurin-9-yl)purine-2-amine | HMDB0260431 | - | M-H | C₁₀H₈N₁₀ | 3 |
| 0.85_300.0684n | Sulfaquinoxaline | HMDB0033139 | - | M-H2O-H, M+Cl | C₁₄H₁₂N₄O₂S | 3 |

Supplementary Table 3. Metabolites identified at the confidence level 3 (continued).

| **Feature** | **Identified lipids** | **HMDB** | **Acquisition** | **Adduct** | **Formula** | **Identification level** |
| --- | --- | --- | --- | --- | --- | --- |
| 29.09_236.9725*m/z* | Benzoyl phosphate | HMDB0060440 | - | M+Cl | C₇H₇O₅P | 3 |
| 6.82_298.9348*m/z* | Mitotane | HMDB0014786 | - | M-H2O-H | C₁₄H₁₀Cl₄ | 3 |
| 11.07_215.0914*m/z* | 5-(4-Carboxybutylperoxy)pentanoic acid | HMDB0260291 | - | M-H2O-H | C₁₀H₁₈O₆ | 3 |
| 12.32_215.0917*m/z* | 5-(4-Carboxybutylperoxy)pentanoic acid | HMDB0260291 | - | M-H2O-H | C₁₀H₁₈O₆ | 3 |
| 17.42_449.1712*m/z* | Abecarnil | HMDB0247747 | - | M+FA-H | C₂₄H₂₄N₂O₄ | 3 |
| 10.05_271.1529*m/z* | N-Methylolpentamethylmelamine | HMDB0255189 | - | M+FA-H | C₉H₁₈N₆O | 3 |
| 13.90_237.0754*m/z* | Arginine chloride | HMDB0248582 | - | M+FA-H | C₆H₁₃ClN₄O | 3 |
| 0.75_869.3341*m/z* | Aclacinomycin X | HMDB0247948 | - | M+FA-H | C₄₂H₅₂N₂O₁₅ | 3 |
| 6.44_276.1557n | Trimethylsilyl ester of alpha-cyclopentylphenylacetic acid | JP001101 | - | M-H, M+FA-H | C₁₆H₂₄O₂Si | 3 |
| 0.85_162.9770*m/z* | 4-Bromo-2-methylbutenal | JP010942 | - | M-H | C₅H₉BrO | 3 |
| 1.67_160.9979*m/z* | Tetrafluorodiaziridine | JP002970 | - | M+FA-H | CF₄N₂ | 3 |
| 12.06_317.1582*m/z* | S-Japonin | HMDB0035802 | - | M-H2O-H | C₁₉H₂₈O₃S | 3 |
| 10.66_125.0964*m/z* | N(tele)-Methylhistaminium | HMDB0062574 | - | M-H | C₆H₁₂N₃⁺ | 3 |
| 7.76_153.0179*m/z* | Biuret | HMDB0249281 | - | M+Cl | C₂H₆N₄O₂ | 3 |
| 12.95_403.1957*m/z* | 4-Fluorobutyrylfentanyl | HMDB0255010 | - | M+Cl | C₂₃H₂₉FN₂O | 3 |
| 23.17_375.2913*m/z* | Isoallolithocholic acid | HMDB0000713 | - | M-H | C₂₄H₄₀O₃ | *Species level* |
| 0.93_187.9900*m/z* | Quinoclamine | MSJ00249 | - | M-H2O-H | C₁₀H₆ClNO₂ | 3 |
| 7.02_216.0945*m/z* | 2-Dimethylamino-4-(4-fluorophenyl)pyrimidine | JP004476 | - | M-H | C₇H₇O₅P | 3 |
| 8.59_197.1175*m/z* | Undecanoic acid | HMDB0000888 | - | M-H2O-H | C₁₄H₁₀Cl₄ | *Species level* |
| 9.86_195.1017*m/z* | Isobutyl 2-furanpropionate | HMDB0037734 | - | M-H | C₁₀H₁₈O₆ | 3 |

Supplementary Table 3. Metabolites identified at the confidence level 3 (continued).

| **Feature** | **Identified lipids** | | **HMDB** | **Acquisition** | | **Adduct** | | **Formula** | | **Identification level** |
| --- | --- | --- | --- | --- | --- | --- | --- | --- | --- | --- |
| 8.46_331.0335*m/z* | N-Methoxyspirobrassinol methyl ether | | HMDB0041059 | - | | M+Cl | | C₁₃H₁₆N₂O₂S₂ | | 3 |
| 12.12_201.1115*m/z* | 1,3,4-Trimethyl-1-phenyl-1-silacyclopent-3-ene | | JP011688 | - | | M-H | | C₁₃H₁₈Si | | 3 |
| 9.54_167.0704*m/z* | (3-Aminopropoxy)guanidine | | HMDB0039228 | - | | M+Cl | | C₄H₁₂N₄O | | 3 |
| 0.81_279.9546*m/z* | [Nitrilotris(methylene)]triphosphonic acid | | HMDB0029807 | - | | M-H2O-H | | C₃H₁₂NO₉P₃ | | 3 |
| 9.40_259.1537*m/z* | 3-(Dimethylfluorosilyl)-3,7-dimethyl-1,6-octadiene | | JP004767 | - | | M+FA-H | | C₁₂H₂₃FSi | | 3 |
| 7.30_250.0839n | Citrinin | | HMDB0041857 | + | | M+Na, M+K, M+H | | C₁₃H₁₄O₅ | | 3 |
| 3.06_173.0803*m/z* | 3-Hydroxysuberic acid | | HMDB0000325 | + | | M+H-H2O | | C₈H₁₄O₅ | | 3 |
| 21.22_209.1153*m/z* | 3-Oxodecanoic acid | | HMDB0010724 | + | | M+Na | | C₁₀H₁₈O₃ | | 3 |
| 28.02_229.2402n | Xestoaminol C | | HMDB0304813 | + | | M+H, M+Na | | C₁₄H₃₁NO | | 3 |
| 22.43_83.0214*m/z* | Urea | | HMDB0000294 | + | | M+Na | | CH₄N₂O | | 3 |
| 11.54_231.0763*m/z* | N-(4-Aminobenzoic acid)-L-glutamic acid | | HMDB0255007 | + | | M+H-2H2O | | C₁₂H₁₄N₂O₅ | | 3 |
| 10.61_221.0787*m/z* | Ethylene glycol dimethacrylate | | HMDB0252072 | + | | M+Na | | C₁₀H₁₄O₄ | | 3 |
| 0.82_217.0691*m/z* | Caffeine | | HMDB0001847 | + | | M+Na | | C₈H₁₀N₄O₂ | | 3 |
| 6.12_198.0378*m/z* | N-Acetyl-L-aspartic acid | | HMDB0000812 | + | | M+Na | | C₆H₉NO₅ | | 3 |
| 0.86_191.0176*m/z* | Uric acid | | HMDB0000289 | + | | M+Na | | C₅H₄N₄O₃ | | 3 |
| 5.42_144.1027*m/z* | Bis(trimethylsilyl)amine | | JP004666 | + | | M+H-H2O | | C₆H₁₉NSi₂ | | 3 |
| 5.80_165.0907*m/z* | 2-Phenylbutyric acid | | HMDB0000329 | + | | M+H | | C₁₀H₁₂O₂ | | 3 |
| 23.14_113.1325*m/z* | Octanol | | HMDB0001183 | + | | M+H-H2O | | C₈H₁₈O | | 3 |
| 5.95_269.0992*m/z* | Trimethyl 1,3,5-pentanetricarboxylic acid ester | | JP004232 | + | | M+Na | | C₁₁H₁₈O₆ | | 3 |
| 1.17_184.0231n | 5-Hydroxyisourate | | HMDB0030097 | + | | M+H, M+K | | C₅H₄N₄O₄ | | 3 |
| 9.16_192.1232*m/z* | N-(2,3-Dihydroxypropyl)valine | | HMDB0254994 | + | | M+H | | C₈H₁₇NO₄ | | 3 |
| 0.75_1012.7940*m/z* | PC 49:2 | LipidBlast061515 | | | - | | M+FA-H | C₅₇H₁₁₀NO₈P | *Species level* | |
| 25.79_257.0417*m/z* | PA 4:0 | LipidBlast459014 | | | + | | M+H | C₇H₁₃O₈P | *Species level* | |

Supplementary Table 3. Metabolites identified at the confidence level 3 (continued).

| **Feature** | **Identified lipids** | **HMDB** | **Acquisition** | **Adduct** | **Formula** | **Identification level** |
| --- | --- | --- | --- | --- | --- | --- |
| 17.28_197.1534*m/z* | 3-Oxododecanoic acid | HMDB0010727 | + | M+H-H2O | C₁₂H₂₂O₃ | *species level* |
| 12.63_173.1645*m/z* | 4-Amino-2,2,6,6-tetramethylpiperidin-1-ol | HMDB0246342 | + | M+H | C₉H₂₀N₂O | 3 |
| 17.23_283.2261*m/z* | Heptadecanedioic acid | LMFA01170028 | + | M+H-H2O | C₁₇H₃₂O₄ | *species level* |
| 8.81_219.0430*m/z* | Daidzein | HMDB0003312 | + | M+H-2H2O | C₁₅H₁₀O₄ | 3 |
| 18.21_235.2052*m/z* | 3-Oxohexadecanoic acid | HMDB0010733 | + | M+H-2H2O | C₁₆H₃₀O₃ | *species level* |
| 14.43_255.1211*m/z* | n-Propyl-L-arginine | HMDB0255235 | + | M+K | C₉H₂₀N₄O₂ | 3 |
| 3.96_109.0050*m/z* | Ethyl phosphate | HMDB0012228 | + | M+H-H2O | C₂H₇O₄P | 3 |
| 11.70_261.1689*m/z* | (x)-2-Heptanol glycoside | HMDB0035028 | + | M+H-H2O | C₁₃H₂₆O₆ | 3 |
| 16.67_641.2856*m/z* | PG 24:4 (Phosphatidylglycerol 24:4) | LipidBlast064900 | + | M+K | C₃₀H₅₁O₁₀P | *species level* |
| 7.24_231.1582*m/z* | Dodecanedioic acid | HMDB0000623 | + | M+H | C₁₂H₂₂O₄ | 3 |
| 7.95_275.0715*m/z* | Hoslundal | LMPK12110172 | + | M+H-2H2O | C₁₈H₁₄O₅ | 3 |
| 16.44_89.0595*m/z* | Butyric acid | HMDB0000039 | + | M+H | C₄H₈O₂ | 3 |
| 16.00_235.0689*m/z* | 2-[4-(Dimethylamino)phenyl]benzothiazol-6-ol | HMDB0250064 | + | M+H-2H2O | C₁₅H₁₄N₂OS | 3 |
| 10.71_245.0990*m/z* | Isopropyl beta-D-glucoside | HMDB0032705 | + | M+Na | C₉H₁₈O₆ | 3 |
| 0.82_119.0583n | Threonine | HMDB0000167 | + | M+H-H2O, M+Na | C₄H₉NO₃ | 3 |
| 1.43_141.0162*m/z* | Erythrono-1,4-lactone | HMDB0000349 | + | M+Na | C₄H₆O₄ | 3 |
| 0.82_241.0306*m/z* | Cystine | HMDB0250712 | + | M+H | C₆H₁₂N₂O₄S₂ | 3 |
| 8.33_192.1236*m/z* | N-(2,3-Dihydroxypropyl)valine | HMDB0254994 | + | M+H | C₈H₁₇NO₄ | 3 |
| 16.67_253.0898n | 10-Azabenzo[a]pyrene | SM828302 | + | M+H-H2O, M+H | C₁₉H₁₁N | 3 |
| 29.01_336.8570*m/z* | 1,2,3,4,6-Pentachlorodibenzofuran | HMDB0244126 | - | M-H | C₁₂H₃Cl₅O | 3 |

Supplementary Table 3. Metabolites identified at the confidence level 3 (continued).

| **Feature** | **Identified lipids** | **HMDB** | **Acquisition** | **Adduct** | **Formula** | **Identification level** |
| --- | --- | --- | --- | --- | --- | --- |
| 10.01_417.2109*m/z* | MGDG 11:0 | LipidBlast057861 | - | M-H2O-H | C₂₀H₃₆O₁₀ | *species level* |
| 12.36_357.1907*m/z* | 2-(3,7,11-Trimethyldodeca-2,6,10-trienylsulfanyl)benzoic acid | HMDB0244927 | - | M-H | C₂₂H₃₀O₂S | 3 |
| 20.65_162.9119*m/z* | Calcium oxalate | HMDB0302182 | - | M+Cl | C₂CaO₄ | 3 |
| 9.62_239.1278*m/z* | Avenolide | LMFA07040176 | - | M-H | C₁₃H₂₀O₄ | 3 |
| 1.21_286.9700*m/z* | 2,7-Naphthalenedisulfonic acid | EA065451 | - | M-H | C₁₀H₈O₆S₂ | 3 |
| 10.47_365.1800*m/z* | Leucomalachite green | HMDB0254033 | - | M+Cl | C₂₃H₂₆N₂ | 3 |
| 9.22_345.0257*m/z* | Xanthylic acid | HMDB0001554 | - | M-H2O-H | C₁₀H₁₃N₄O₉P | 3 |
| 1.20_287.0620*m/z* | Sulfomethylmethane | HMDB0258597 | - | M+FA-H | C₈H₁₈O₄S₂ | 3 |
| 13.96_287.1849*m/z* | Arginine ornithine | HMDB0248583 | - | M-H | C₁₁H₂₄N₆O₃ | 3 |
| 8.62_255.1227*m/z* | Canangalin I | LMPR0103010028 | - | M-H | C₁₃H₂₀O₅ | 3 |
| 8.97_345.0483*m/z* | Glucose isomerase from Streptomyces rubiginosus | HMDB0032295 | - | M-H2O-H | C₁₇H₁₇ClN₂O₃S | 3 |
| 7.86_307.1368*m/z* | Streptidine | HMDB0258506 | - | M+FA-H | C₈H₁₈N₆O₄ | 3 |
| 10.85_303.1068*m/z* | Finrozole | HMDB0252263 | - | M-H2O-H | C₁₈H₁₅FN₄O | 3 |
| 0.85_170.0239n | 4-Chlorophenylurea | LU129801 | - | M-H2O-H, M+Cl | C₇H₇ClN₂O | 3 |
| 6.17_175.0965*m/z* | 3-Acetyl-1-trideuteromethylindole | JP000447 | - | M-H | C₁₁H₂₃NO | 3 |
| 14.70_201.1486*m/z* | 3-Hydroxynonyl acetate | HMDB0032443 | - | M-H | C₁₁H₂₂O₃ | *species level* |

Notes: Compounds are displayed as Retention time (RT) measured m/z values or RT neutral mass when denoted by an ‘n’. Libraries used for identification included LipidMaps, the Human Metabolome Database (HMDB), and libraries available in MoNA - MassBank of North America. “–” indicates negative ionization mode and “+” indicates positive ionization mode.

Supplementary Table 4. Metabolites identified at the confidence level 2.

| **Feature** | **Identified metabolites** | **HMDB** | **Acquisition mode** | **Adduct** | **Chemical Formula** | **Identification level** |
| --- | --- | --- | --- | --- | --- | --- |
| 1.20_335.0476*m/z* | ((2-Amino-3-((2-amino-3-((carboxymethyl) amino)-3- oxopropyl)dithio)propanoyl) amino)acetic acid | HMDB0242125 | - | M-H2O-H | C₁₀H₁₈N₄O₆S₂ | 2 |
| 10.03_311.1117*m/z* | 5'-Amino-5'-deoxyadenosine | HMDB0246876 | - | M+FA-H | C₁₀H₁₄N₆O₃ | 2 |
| 1.21_264.9875*m/z* | ribose-5-phosphate | HMDB0257215 | - | M+Cl | C₅H₁₁O₈P | 2 |
| 10.84_271.1535*m/z* | N-Methylolpentamethylmelamine | HMDB0255189 | - | M+FA-H | C₉H₁₈N₆O | 2 |
| 12.33_223.0961*m/z* | Homoarginine | HMDB0000670 | - | M+Cl | C₇H₁₆N₄O₂ | 2 |
| 16.84_378.2395*m/z* | Phytosphingosine-1-P | HMDB0012280 | - | M-H2O-H | C₁₈H₄₀NO₆P | 2 |
| 9.96_200.0919*m/z* | Pantothenic acid | HMDB0000210 | - | M-H2O-H | C₉H₁₇NO₅ | 2 |
| 9.99_383.0575*m/z* | Propanoic acid. 3-((((2-(1.1-dimethylethyl)- 5-methoxy-6- benzothiazolyl)amino)thioxomethyl)thio)- | HMDB0249848 | - | M-H | C₁₆H₂₀N₂O₃S₃ | 2 |
| 1.32_259.1276*m/z* | isoleucine glutamate | HMDB0253650 | + | M+H-H2O | C₁₁H₂₀N₂O₆ | 2 |
| 10.12_220.0636*m/z* | N-Acetylalliin | HMDB0242184 | + | M+H | C₈H₁₃NO₄S | 2 |
| 11.06_203.1162*m/z* | Acetylcarnitine | HMDB0000201 | + | M+H-H2O, M+H | C₉H₁₇NO₄ | 2 |
| 11.49_148.1120*m/z* | Ethanone. 1-(9-azabicyclo(4.2.1)non-2-en-2-yl)-. (1R)- | HMDB0030346 | + | M+H | C₁₀H₁₃N | 2 |

Supplementary Table 4. Metabolites identified at the confidence level 2 (continued).

| **Feature** | **Identified metabolites** | **HMDB** | **Acquisition** | **Adduct** | **Formula** | **Identification level** |
| --- | --- | --- | --- | --- | --- | --- |
| 17.17_414.2021*n* | LPG 11:1 | LipidBlast454428 | + | M+H-H2O, M+H. M+Na, M+K | C₁₇H₃₅O₉P | 2 |
| 16.00_432.2373*m/z* | Pentadeca-4.6.8-trienedioylcarnitine | HMDB0241452 | + | M+Na | C₂₂H₃₅NO₆ | 2 |
| 17.23_227.0833*m/z* | 2-tridecene-4.7-diynal | LMFA06000077 | + | M+K | C₁₃H₁₆O | 2 |
| 18.39_263.1275*m/z* | 13-Hydroxyabscisic acid | HMDB0036095 | + | M+H-H2O | C₁₅H₂₀O₅ | 2 |
| 2.41_146.0577*m/z* | Adipic acid | HMDB0000448 | + | M+H. M+Na | C₆H₁₀O₄ | 2 |
| 2.41_164.0914*m/z* | Glucamine | HMDB0246693 | + | M+H-H2O | C₆H₁₅NO₅ | 2 |
| 2.75_209.0917*m/z* | Porphobilinogen | HMDB0000245 | + | M+H-H2O | C₁₀H₁₄N₂O₄ | 2 |
| 22.59_376.2617*n* | MG 20:5 | HMDB0011550 | + | M+H. M+Na, M+K | C₂₃H₃₆O₄ | 2 |
| 23.14_454.2921*m/z* | 3-Hydroxyhexadecanoylcarnitine | HMDB0013336 | + | M+K | C₂₃H₄₅NO₅ | 2 |
| 23.59_404.2927*n* | MG 22:5 | HMDB0011555 | + | M+H. M+Na | C₂₅H₄₀O₄ | 2 |
| 3.92_219.1336*m/z* | Carboxyethyllysine | HMDB0028916 | + | M+H | C₉H₁₈N₂O₄ | 2 |
| 6.75_147.0651*m/z* | 2-Methylglutaric acid | HMDB0000422 | + | M+H | C₆H₁₀O₄ | 2 |
| 8.79_206.0818*m/z* | N-Acetyl tyrosine | HMDB0000866 | + | M+H-H2O | C₁₁H₁₃NO₄ | 2 |
| 9.26_168.0418*n* | Homogentisic acid | HMDB0000130 | + | M+H-H2O, M+Na. M+H | C₈H₈O₄ | 2 |

Supplementary Table 4. Metabolites identified at the confidence level 2 (continued).

| **Feature** | **Identified metabolites** | **HMDB** | **Acquisition** | **Adduct** | **Formula** | **Identification level** |
| --- | --- | --- | --- | --- | --- | --- |
| 10.15_365.1799*m/z* | (2R.6x)-7-Methyl-3-methylene-1.2.6.7-octanetetrol 2-glucoside | HMDB0033216 | - | M-H | C₁₆H₃₀O₉ | 2 |
| 9.07_388.2079*n* | 9.13-Dihydroxy-4-megastigmen-3-one 9-glucoside | HMDB0036318 | **-** | M+Cl, M+FA-H | C₁₉H₃₂O₈ | 2 |
| 13.57_241.1433*m/z* | 10-(2.3-Dihydroxypropoxy)-10-oxodecanoic acid | HMDB0257660 | + | M+H-2H2O | C₁₃H₂₄O₆ | 2 |
| 13.46_269.1379*m/z* | Arginine butyl ester | CCMSLIB00005723439 | + | M+K | C₁₀H₂₂N₄O₂ | 2 |
| 20.27_246.2192*n* | 8.8-Diethoxy-2.6-dimethyl-2-octanol | HMDB0034557 | + | M+H-H2O, M+H. M+H-2H2O | C₁₄H₃₀O₃ | 2 |
| 19.31_249.1027*m/z* | Sudan I | HMDB0258557 | + | M+H | C₁₆H₁₂N₂O | 2 |
| 11.49_334.1492*m/z* | 3.5-DIPHENYL-2.4.7-OCTANETRIONE (1.1.1-D3) | JP005539 | + | M+Na | C₂₀H₂₃O₃ | 2 |
| 1.81_193.0292*m/z* | 2.5-Dimethyl-3-furanthiol acetate | HMDB0032234 | + | M+Na | C₈H₁₀O₂S | 2 |

Supplementary Table 4. Metabolites identified at the confidence level 2 (continued).

| **Feature** | **Identified metabolites** | **HMDB** | **Acquisition** | **Adduct** | **Formula** | **Identification level** |
| --- | --- | --- | --- | --- | --- | --- |
| 18.16_251.1273*m/z* | Mifentidine | HMDB0254714 | + | M+Na | C₁₃H₁₆N₄ | 2 |
| 9.67_392.2273*m/z* | Valyl-prolyl-glycyl-valyl-glycine | HMDB0259877 | + | M+H-2H2O | C₁₉H₃₃N₅O₆ | 2 |
| 11.54_294.1453*m/z* | Phenylalanylglutamine | HMDB0028804 | + | M+H | C₁₄H₁₉N₃O₄ | 2 |
| 16.00_414.2031*m/z* | 4-O-Methylmelleolide | HMDB0037039 | + | M+Na. M+K | C₂₄H₃₀O₆ | 2 |
| 9.26_189.1003*n* | N-Lactoylvaline | HMDB0062181 | + | M+H-H2O, M+H | C₈H₁₅NO₄ | 2 |
| 9.46_286.2008*m/z* | 2-Octenoylcarnitine | HMDB0013324 | + | M+H | C₁₅H₂₇NO₄ | 2 |
| 8.80_231.0768*m/z* | Indole-3-acetylglycine | HMDB0240661 | **-** | M-H | C₁₇H₃₅O₉P | 2 |
| 11.67_275.1024*m/z* | N-lactoyl-Tryptophan | HMDB0062178 | **-** | M-H | C₂₂H₃₅NO₆ | 2 |

Notes: Compounds are displayed as Retention time (RT) measured *m/z* values or RT neutral mass when denoted by an ‘n’. Libraries used for identification included LipidMaps, the Human Metabolome Database (HMDB), and libraries available in MoNA - MassBank of North America. “–” indicates negative ionization mode and “+” indicates positive ionization mode.

**Supplementary Table 5. Lipids identified at the confidence level 3.**

| **Feature** | **Identified lipids** | **HMDB** | **Acquisition** | **Adduct** | **Formula** | **Identification level** |
| --- | --- | --- | --- | --- | --- | --- |
| 0.85_194.9877*m/z* | Pentafluorobenzaldehyde | HMDB0256257 | - | M-H | C₇HF₅O | *Species level* |
| 39.28_246.9762*m/z* | Calcium galactarate | HMDB0303726 | - | M-H | C₆H₈CaO₈ | *Species level* |
| 10.19_97.0291*m/z* | Levulinic acid | HMDB0000720 | - | M-H2O-H | C₅H₈O₃ | 3 |
| 22.28_129.0922*m/z* | Heptanoic acid | HMDB0000666 | - | M-H | C₇H₁₄O₂ | 3 |
| 18.71_297.1529*m/z* | C10LAS (Linear alkylbenzene sulfonate with C10 chain) | LIT00037 | - | M-H | C₁₆H₂₆O₃S | *Species level* |
| 7.54_579.2633*m/z* | Rupintrivir | HMDB0257363 | - | M-H2O-H | C₃₁H₃₉FN₄O₇ | *Species level* |
| 39.13_243.9091n | 2,3,5-Trichloro-cis,cis-muconic acid | LMFA01090150 | - | M-H, M+FA-H | C₆H₃Cl₃O₄ | *Species level* |
| 1.98_301.1284*m/z* | Octanoyl glucuronide | HMDB0010347 | - | M-H2O-H | C₁₄H₂₄O₈ | 3 |
| 10.14_694.3706n | PI 24:2 (Phosphatidylinositol 24:2) | LipidBlast470109 | - | M-H2O-H, M-H | C₃₃H₅₉O₁₃P | *Species level* |
| 16.48_71.0854*m/z* | Isopentanol | HMDB0006007 | + | M+H-H2O | C₅H₁₂O | 3 |
| 8.07_179.0104*m/z* | 4,6-Dinitro-o-cresol | HMDB0245221 | - | M-H2O-H | C₇H₆N₂O₅ | 3 |

Notes: Compounds are displayed as Retention time (RT) measured m/z values or RT neutral mass when denoted by an ‘n’. Libraries used for identification included LipidMaps, the Human Metabolome Database (HMDB), and libraries available in MoNA - MassBank of North America. “–” indicates negative ionization mode and “+” indicates positive ionization mode.

Supplementary Table 6. Lipids identified at the confidence level 2.

| **Feature** | **Identified lipids** | **HMDB** | **Acquisition** | **Adduct** | **Formula** | **Identification level** |
| --- | --- | --- | --- | --- | --- | --- |
| 20.85_568.3609*m/z* | LysoPC 18:0 | HMDB0010384 | - | M+FA-H | C₂₆H₅₄NO₇P | Species levels |
| 20.04_330.2772*n* | MG 16:0 | HMDB0011533 | - | M-H, M+FA-H, M+Cl | C₁₉H₃₈O₄ | Species levels |
| 13.67_274.2138*n* | Turpetholic acid A | LMFA01050184 | - | M-H, M+FA-H | C₁₅H₃₀O₄ | Species levels |
| 22.23_394.1537*m/z* | Pro-Pro-Phe | HMDB0304811 | - | M+Cl | C₁₉H₂₅N₃O₄ | Species levels |
| 15.93_488.2992*m/z* | Glycocholic acid | HMDB0000138 | + | M+Na | C₂₆H₄₃NO₆ | Species levels |
| 22.61_465.4283*m/z* | FAHFA 16:0/9O(FA 14:0) | LMFA07090097 | + | M+H-H2O | C₃₀H₅₈O₄ | Species levels |
| 14.35_337.2724*m/z* | Pregnanetriol | HMDB0006070 | + | M+H | C₂₁H₃₆O₃ | Species levels |
| 19.25_406.2703*n* | Alfaprostolum | HMDB0248132 | + | M+H-H2O, M+H | C₂₄H₃₈O₅ | Species levels |
| 8.74_330.1453*m/z* | Prolinamide,glycyl-N-(4- methyl-2-oxo-2H-1-benzopyran-7-yl)- | HMDB0252834 | + | M+H | C₁₇H₁₉N₃O₄ | Species levels |
| 7.22_284.2209*m/z* | C12-homoserine lactone | RP020503 | + | M+H | C₁₆H₂₉NO₃ | Species levels |
| 12.84_202.1795*m/z* | 11-amino-undecanoic acid | LMFA01100004 | + | M+H | C₁₁H₂₃NO₂ | Species levels |
| 11.97_348.2367*m/z* | 3,8- Dihydroxydecanoylcarnitine | HMDB0241141 | + | M+H | C₁₇H₃₃NO₆ | Species levels |
| 18.52_277.2167*m/z* | 2R-hydroxy-linoleic acid | LMFA02000057 | - | M-H2O-H | C₁₈H₃₂O₃ | Species levels |

Supplementary Table 6. Lipids identified at the confidence level 2 (continued).

| **Feature** | **Identified lipids** | **HMDB** | **Acquisition** | **Adduct** | **Formula** | **Identification level** |
| --- | --- | --- | --- | --- | --- | --- |
| 11.99_478.2102*m/z* | 2-(2-Benzoylanilino)-3-[4-[2-[methyl(2-pyridinyl)amino]ethoxy]phenyl]propanoic acid | HMDB0252979 | + | M+H-H2O | C₃₀H₂₉N₃O₄ | Species levels |
| 9.21_277.1788*m/z* | Gingerol | HMDB0005783 | + | M+H-H2O | C₁₇H₂₆O₄ | Species levels |
| 17.65_672.3406*m/z* | Cycloaspeptide H_130057 | CCMSLIB00000478491 | + | M+H | C₃₇H₄₅N₅O₇ | Species levels |
| 9.38_459.2605*m/z* | [6]-Gingerdiol 4'-O-beta-D-glucopyranoside | HMDB0036122 | + | M+H | C₂₃H₃₈O₉ | Species levels |
| 9.57_454.2176*m/z* | puromycin | HMDB0256926 | + | M+H-H2O | C₂₂H₂₉N₇O₅ | Species levels |
| 9.05_407.2027*m/z* | 20-Trihydroxy-leukotriene-B4 | HMDB0012643 | + | M+Na | C₂₀H₃₂O₇ | Species levels |
| 15.06_316.2832*m/z* | Dehydrophytosphingosine | HMDB0038057 | + | M+H | C₁₈H₃₇NO₃ | Species levels |
| 16.89_440.1503*m/z* | artemisone | HMDB0248624 | + | M+K | C₁₉H₃₁NO₆S | Species levels |
| 10.57_593.2803*m/z* | DGDG 12:0 | LipidBlast407275 | - | M-H2O-H | C₂₇H₄₈O₁₅ | Species levels |
| 8.72_515.1211*m/z* | Tarenninoside E | LMPR0102070048 | - | M-H2O-H | C₂₅H₂₆O₁₃ | Species levels |
| 3.78_227.1648*m/z* | 2-Dodecenal | HMDB0031020 | - | M+FA-H | C₁₂H₂₂O | Species levels |

Notes: Compounds are displayed as Retention time (RT) measured *m/z* values or RT neutral mass when denoted by an ‘n’. Libraries used for identification included LipidMaps, the Human Metabolome Database (HMDB), and libraries available in MoNA - MassBank of North America. “–” indicates negative ionization mode and “+” indicates positive ionization mode.

**Online Resource 2 – Supplementary figures**


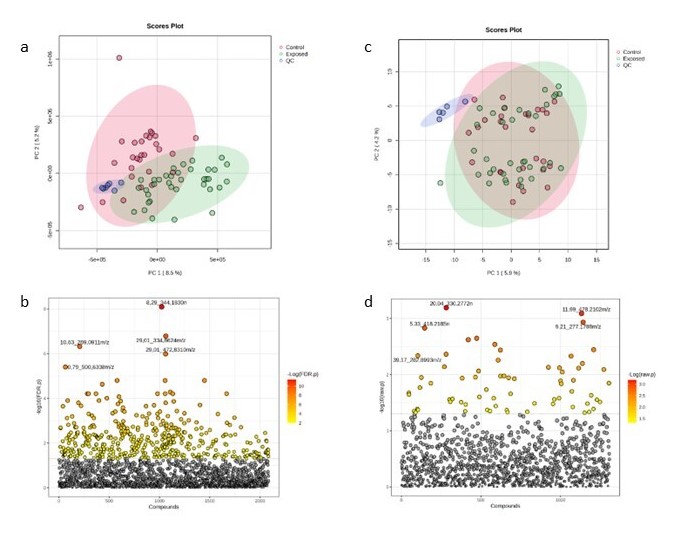


**Supplementary** **Fig. 1** score chart of Principal Component Analysis (PCA) and student t test. Left panel (A-B): results for metabolites. Right panel (C-D): Results for lipids. (A. C) Score chart of Principal Component Analysis (PCA); (B. D) Student's t-test with an adjusted p-value (FDR) ≤ 0.05.


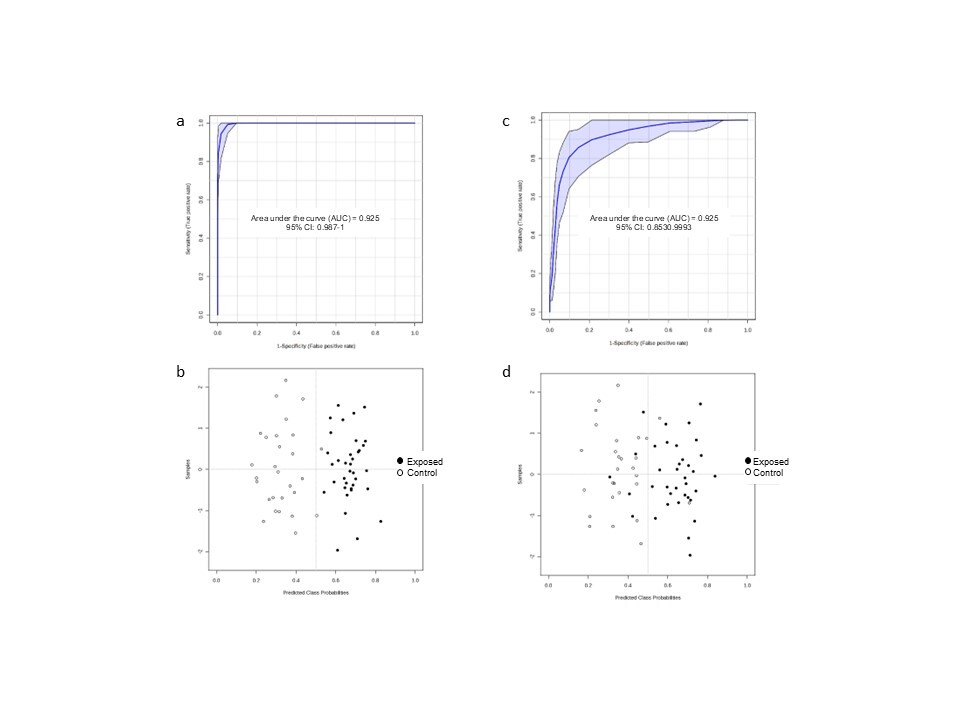


**Supplementary Fig. 2** ROC curves and confusion matrices obtained using the PLS-DA method analysis. Left panel (A-B): results for 190 identified metabolites. Right panel (C-D): results for 34 identified lipids; (A, C) ROC curve with 95% confidence interval (CI) for the model generated, showing an AUC score of 1.0 and 0.935, respectively; (B, D) Confusion matrix of predicted group probabilities.
